# Fatty Acids: An Insight into the Pathogenesis of Neurodegenerative Diseases and Therapeutic Potential

**DOI:** 10.3390/ijms23052577

**Published:** 2022-02-25

**Authors:** Diego Julián Vesga-Jiménez, Cynthia Martin, George E. Barreto, Andrés Felipe Aristizábal-Pachón, Andrés Pinzón, Janneth González

**Affiliations:** 1 Departamento de Nutrición y Bioquímica, Facultad de Ciencias, Pontificia Universidad Javeriana, Bogota 110231, Colombia; vesgad@javeriana.edu.co (D.J.V.-J.); andres_aristizabal@javeriana.edu.co (A.F.A.-P.); 2 Division of Neuropharmacology and Neurologic Diseases, Yerkes National Primate Research Center, Atlanta, GA 30329, USA; cmart51@emory.edu; 3 Department of Biological Sciences, University of Limerick, V94 T9PX Limerick, Ireland; george.barreto@ul.ie; 4 Health Research Institute, University of Limerick, V94 T9PX Limerick, Ireland; 5 Laboratorio de Bioinformática y Biología de Sistemas, Universidad Nacional de Colombia, Bogota 111321, Colombia; ampinzonv@unal.edu.co

**Keywords:** palmitic acid, brain cells, neurodegenerative diseases, inflammation, fatty acids, neuroprotection

## Abstract

One of the most common lipids in the human body is palmitic acid (PA), a saturated fatty acid with essential functions in brain cells. PA is used by cells as an energy source, besides being a precursor of signaling molecules and protein tilting across the membrane. Although PA plays physiological functions in the brain, its excessive accumulation leads to detrimental effects on brain cells, causing lipotoxicity. This mechanism involves the activation of toll-like receptors (TLR) and nuclear factor kappa-light-chain-enhancer of activated B cells (NF-κB) pathways, with the consequent release of pro-inflammatory cytokines, increased production of reactive oxygen species (ROS), endoplasmic reticulum (ER) stress, and autophagy impairment. Importantly, some of the cellular changes induced by PA lead to an augmented susceptibility to the development of Alzheimer’s and Parkinson´s diseases. Considering the complexity of the response to PA and the intrinsic differences of the brain, in this review, we provide an overview of the molecular and cellular effects of PA on different brain cells and their possible relationships with neurodegenerative diseases (NDs). Furthermore, we propose the use of other fatty acids, such as oleic acid or linoleic acid, as potential therapeutic approaches against NDs, as these fatty acids can counteract PA’s negative effects on cells.

## 1. Introduction

Lipids are key components of the structure and function of the brain [1,2,3]. They can be used as an energy source and participate in different physiological processes, such as cell transport, protein stabilization, cell signaling and transduction, and synaptic transmission, among others [4,5]. Lipids, such as phosphatidylinositol, can act as signaling molecules [6,7] modulating inflammation and other processes, such as cell survival and senescence [2,8]. In the adult brain, most of the lipids are found in the myelin sheaths formed by oligodendrocytes. The myelin sheaths have a higher ratio of lipids (70–80% of its composition) compared with the other membranes in the brain, which are composed of nearly 40% lipids. In all these membranes, the most abundant lipids are glycosphingolipids (GSLs), cholesterol, and phospholipids [9,10]. In the case of the GSLs, they have two roles, first as membrane receptors for an extracellular GSL binding ligand, functioning as antigens or mediators of cell adhesion, and those in which membrane GSLs interact laterally with other components of the cell membrane, particularly growth factor receptors, to modify signal transduction [11].

On the other hand, cholesterol is another lipid of importance in the brain (Table 1) because it is the precursor of steroid hormones, such as testosterone and estrogen, which exert protective effects and modulate several functions in the brain [12]. Additionally, it has been shown that cholesterol interacts with phospholipids to influence their behavior [13]. Phospholipids can act as signaling molecules and have been implicated in membrane fluidity along with fatty acids (FAs) (Table 1) [2]. Importantly, FAs are the building blocks of phospholipids and are classified as unsaturated (monounsaturated (MUFAs), polyunsaturated (PUFAs), and saturated fatty acids (SAFAs) [14]. PA is the most common and predominant SAFA found in the human body and serves as energy storage. PA is also involved in the regulation of membrane fluidity and the location of transmembrane proteins through the process of palmitoylation [15]. Some studies using different rat tissues reported that incubation with PA induced intracellular inflammatory signaling, mitochondrial dysfunction, and insulin resistance, showing the importance of its balance in human cells [15,16].

Linked to this, it is known that a higher intake of PA for long periods, as observed in obesity, generates metabolic impairment and a set of pathological mechanisms known as lipotoxicity [17,18,19] that causes an increase in the inflammatory response of different cells. In the brain, lipotoxicity causes microglial and astrocytic activation, ceramide formation, oxidative stress (OS), and ER stress, [20,21,22]. There is evidence showing these hallmarks are related to neurodegenerative pathophysiology, for instance, in Alzheimer’s disease (AD) and mild cognitive impairment (MCI) [23]. Interestingly, several studies analyzing lipids in the frontal and parietal cortex tissues of animals and humans suggest that an increase in PA content could have a role in the neurodegenerative disease [24,25], cognitive decline, and brain atrophy caused by excessive inflammation [26].

Previous works indicated PA induces a pro-inflammatory microenvironment, which is critical to damaging blood-brain barrier (BBB) integrity [27,28]. In fact, after BBB breakdown, PA easily crosses this physical barrier, resulting in an increase in its concentration in the brain, promoting harmful and deleterious effects to neurons and glial cells [27,28,29,30]. However, the whole process that triggers PA-induced damage in the brain, the mechanisms underlying this phenomenon, and its relationship with ND progression are not fully understood. In vitro studies suggested that PA induces tau hyperphosphorylation and β-amyloid protein (Aβ) formation, as well as the promotion of mitogen-activated protein kinases (MAPK) and the nuclear translocation of NF-κB in response to oxidative stress in cortical neurons and astrocytes [31,32,33]. PA also increases α-synuclein (ASN) accumulation, which is a signal related to Parkinson’s disease (PD) [34]. Despite these data suggesting that there is a relation between PA and ND pathogenesis, the whole explanation of this association is unknown.

**Figure 1:**
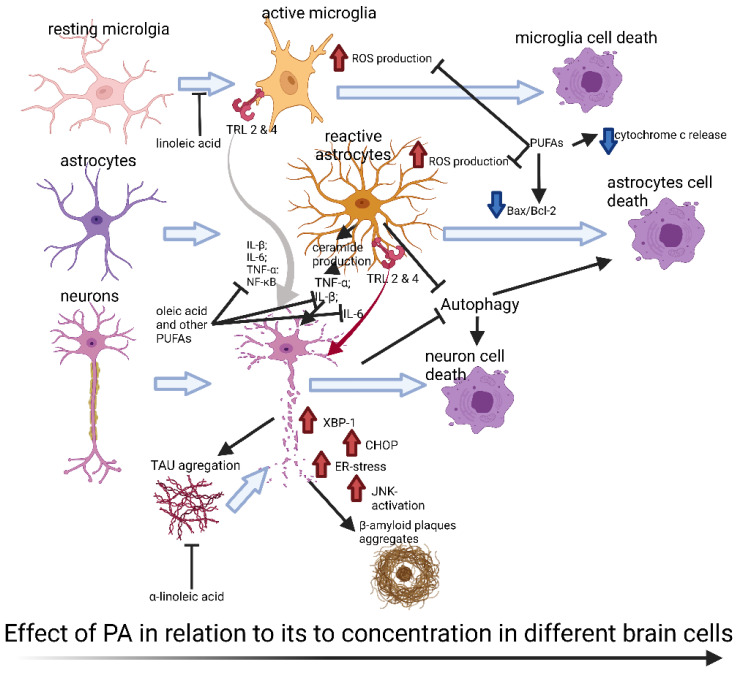
Graphical abstract. This figure shows some of the most reported mechanisms of damage in the different cells of the brain and how it affects neurons, revealing that neurons are prone to cell death due to an increase in the concentration of PA, followed by microglia, and the most resistant cells to PA are the astrocytes, which require higher concentrations of PA to induce cell death. Additionally, this figure shows that, at concentrations where neurons are damaged, astrocytes and microglia are turning more reactive, favoring the production of ROS, ceramides, and pro-inflammatory cytokines, among other pathways that will be explained throughout the manuscript, that will lead to positive feedback, accelerating the damage to the neurons, and promote the formation of the hallmarks of NDs in neurons. The figure also shows the modulation of these deleterious effects using other fatty acids, such as PUFAs, that showed promissory responses for attenuating the damage caused by PA, reducing the inflammatory response, ROS production, cell death, and the induction of pro-apoptotic pathways. Created with BioRender.com.

Given the lack of effective treatments for NDs and the deleterious effects of PA, there is a growing need for applied research focused on new alternatives to treat these conditions [35,36,37]. Some FAs, such as the unsaturated FAs, and the small chain SAFAs, have been shown to ameliorate the damage caused by PA accumulation in the brain [38,39,40]. For instance, unsaturated FAs reduced inflammatory responses, ROS production, palmitate-induced cytotoxicity, and cell death in different models of brain damage induced by PA [38,40,41,42,43] (Figure 1). Other kinds of FAs that have shown beneficial and protective effects are the short and medium-chain SAFAs, including sodium butyrate (NaB), which induces ketogenesis [44] and ameliorates damage in models of AD and Huntington’s disease [45,46]. For this reason, our main aim in this review is to highlight the reported PA mechanisms that trigger detrimental effects in the brain, contrast the evidence found on its different types of cells, discuss the existing evidence of the relationship with NDs, establish the essential role of PA in these diseases, and to offer some insights in possible therapeutic alternatives using other kinds of FAs.

**Table 1:** The role of the different lipids in the brain. A summary list of five of the most common lipid types in biological organisms, triacylglycerols (TAGs), sterol lipids, sphingolipids, phospholipids, and fatty acids, and their functions in the brain.

Lipid	Function in the Brain	Reference
TAGs	Not present in the brain	Hamilton et al., 2007; Tracey et al., 2018 [1,2]
Sterol lipids	Precursor of sex hormones with protective effects in the CNS, such as the estrogens and testosterone	Liu et al., 2010 [12]
	Gives stability and rigidity to the cell membrane and can give thickness in certain areas, helping the formation of lipid rafts	Song et al., 2014 [13]
Sphingolipids	Membrane receptor for an extracellular GSL binding ligand, usually functioning as antigens or mediators of cell adhesion, and membrane GSLs interact laterally with other components of the cell membrane, particularly growth factor receptors	Lingwood et al., 2011; tracey et al., 2018 [2,11]
Phospholipids and Fatty acids	Determine the fluidity of the membrane and have two tails of FAs	Song et al., 2014 [13]
	Determine the ubication of proteins across the membrane, and participate in the formation of lipid rafts	Carta et al., 2017 [15]
	FAs can be used as an energy source	Panov et al., 2014 [4]
	Can act as signaling molecules or as the precursors of signaling molecules, such as phospoinisitol-3 and ceramide	Carta et al., 2017; Kim et al., 2014 [15,27]

## 2. Fatty Acid Roles in Non-Pathological Conditions

This FAs are of vital importance for the brain, since these are part of the cell membrane composition [9,10]. Moreover, these molecules can be used to supply the metabolic demands of the brain [4,47], where about 20% of the total energy required by the brain is met through FA beta-oxidation. It has been suggested that this process is carried out mainly by astrocytes [4,47], although with lower efficiency compared to other tissues and with high energy turnover to avoid excessive OS [44,48]. It has been shown that FAs may regulate neurogenesis, brain vesicular activity, mood, and cognition [38,41]. In this sense, FAs in the brain regulate phosphoinositide 3-kinases (PI3K) [41], peroxisome proliferator-activated receptor (PPAR) [49], G-protein coupled receptors (GPR) [50], protein kinase C (PKC), and NF-κB [51], and all of these are implicated in cell development, growth, energy metabolism, regulation of inflammatory responses and cell survival [52,53,54].

Another important function of FAs is to influence the rigidity of the membrane, making it more flexible when the concentration of unsaturated FAs is increased [55]. Most of the unsaturated FAs in the brain are PUFAs (25–30% of the total FAs in the brain) [2,56], whose presence in all membranes provides more flexibility and stability by increasing the degree of rotation around single bonds [38]. In the brain, they may be regulating the function and structure of endothelial cells, glial cells, and neurons [41]. The most abundant PUFAs in the brain are arachidonic acid (ARA) (ω-6) (8–10% of total FAs), which is a potent signaling molecule in the brain regulating pro-inflammatory responses, and docosahexaenoic acid (DHA) (ω-3) (12–14% of total FAs) [38,41,57]. Importantly, DHA has a role in synaptic integrity and the assembly of the soluble NSF attachment protein receptor (SNARE) complex [58]. It has been shown that DHA attenuates the altered expression of postsynaptic dendritic proteins, including drebrin, postsynaptic density- 95 (PSD95), N-methyl-D-aspartate (NMDA) receptors, and Ca2+–calmodulin-dependent protein kinase 2 (CAMK2) in a mouse model of AD [41,59,60].

Finally, it has been shown that SAFAs contribute to the stabilization of membranes and proteins across the membrane. They could serve as an energy source in the brain and can be the precursors of other signaling molecules [2] (Table 1). It has been shown that PA has a key role in protein distribution across the membrane, which is mediated by a process called palmitoylation [15]. This process will be addressed in the next section.

### Palmitic Acid: Physiological Role in the Brain

PA defines the location of several proteins through the membrane by a process called protein palmitoylation and palmitoylethanolamide (PEA) biosynthesis [15,61,62]. PEA is an endogenous amide that is used as a lipid mediator that exhibits neuroprotective, anti-neuroinflammatory, immunoregulatory, and analgesic activities [15,63,64]. On the other hand, S-palmitoylation is a covalent post-translational modification of proteins, which consists of the reversible addition of PA to specific cysteines via a thioester bond. Its reversibility makes it a form of lipidation, allowing its dynamic regulation [65]. Moreover, S-palmitoylation also allows the fine-tuning of protein functions, such as phosphorylation or ubiquitination [66]. Several mammalian proteins are targets of palmitoylation, many of which are associated with cancer, diabetes, schizophrenia, and neurodegeneration, including AD and Huntington’s disease, among other NDs [67,68,69]. Experimental evidence suggests that palmitoylation acts on the neural cell adhesion molecule (N-CAM) [70,71], CD36 [72], superoxide dismutase (SOD1) [73], leucine-rich glioma-inactivated 1 (LGI1), Niemann–Pick C1 protein (NPC1) that is implicated in AD, the NMDA receptor in the frontal cortex [69,74,75,76,77,78] and chaperones as the presynaptic co-chaperone Cysteine string protein-α (CSPα) [15,79,80].

In addition, palmitoylation has important regulating roles in the central nervous system (CNS), including neurotransmitter release through the 25 kDa synaptosomal-associated protein (SNAP-25a/b). SNAP-25 a/b are SNARE proteins that are highly expressed in the brain and are palmitoylated membrane proteins essential for the exocytosis of neurotransmitters from synaptic terminals [81,82]. Palmitoylation and de-palmitoylation on PSD95,which is the major density protein in the postsynaptic region at excitatory synapses, regulates the number of synaptic α-amino-3-hydroxy-5-methyl-4-isoxazole propionic acid receptors (AMPAR) [83,84]. Therefore, further analysis is necessary for a better understanding of the functions of palmitoylation in the CNS [21].

It has been demonstrated that membrane protein palmitoylation promotes their association with cholesterol and sphingolipid-rich membrane microdomains or lipid rafts [85]. The lipid rafts seem to act as platforms by bringing together various signaling components and facilitating their interaction, such as proteins involved in signal transduction. Palmitoylation can increase the affinity of proteins for these microdomains [15]. Nevertheless, it is not well understood how palmitoylation influences the raft association of proteins. A possible explanation may be the affinity of PA for these lipid domains causing its association and generating the lipid rafts [86].

## 3. Fatty Acid Accumulation, Lipotoxicity, and Brain Dysfunction

**Figure 2:**
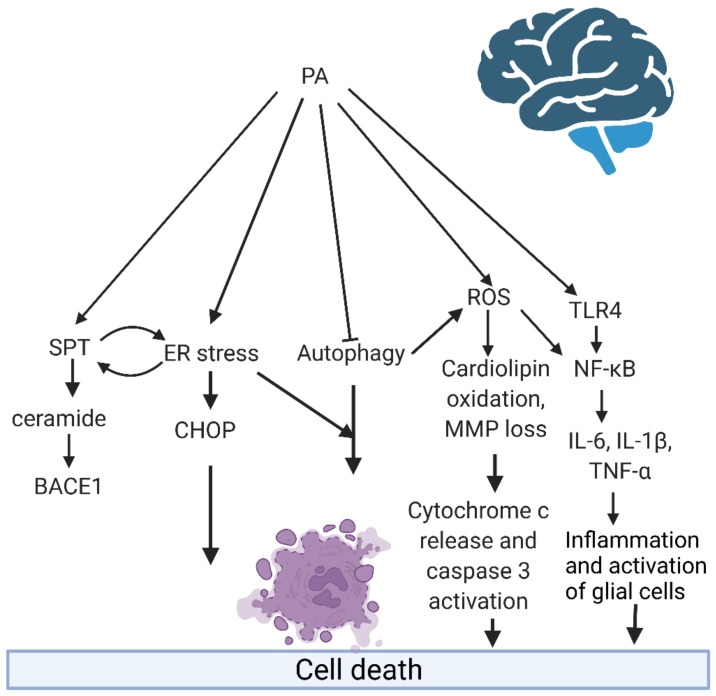
Common mechanisms induced by PA in the brain. Graphical summary of the common pathways in the different brain cells that promote cell damage after the exposition to toxic concentrations of PA explained in Section 4. This figure collects and highlights the common pathways that are reported the most to produce damage in brain cells, showing that the formation of ceramides is also linked to ER stress and the reduction of autophagy that will be derived on the activation of apoptotic pathways and is potentiated by ER stress. Furthermore, autophagy impairment increases ROS production and ROS increase, and the activation of pro-inflammatory pathways are linked. These are the most frequently reported mechanisms that will induce a deleterious effect in the brain. Created with BioRender.com.

Although the homeostatic balance of the lipids in the brain is highly regulated, a long-term high-fat diet (HFD) with a high percent of SAFAs can alter the lipidic profile [87]. The constant exposure of the brain to large amounts of SAFAs, combined with a higher ratio of intake over their oxidation, can cause cellular damage and influence a variety of detrimental metabolic pathways that include inflammation and cell death. This condition is defined as lipotoxicity [17,19,31,88,89] (Figure 2). Lipotoxicity is an allostatic state caused by the constant accumulation of FAs in adipose and non-adipose tissues due to the caloric surplus [19], causing dysfunction and even affecting the survival of cells, tissues, and organs, such as the brain [17,19,31,88,89].

The effects of SAFAs and their accumulation in the brain have been largely reported. Obesity animal models have shown a disruption of BBB integrity, allowing more SAFAs to pass into the brain [28]. Karmi et al., (2010) showed, using an intravenous injection of radiolabeled PA in humans, that this FA can cross the BBB and reach the brain [90]. Within this organ, PA accumulation in astrocytes induces the release of the pro-inflammatory molecules tumor necrotic factor-alpha (TNF-α) and interleukin (IL)-6 [91]. It is speculated that these two cytokines may be two of the main factors that cause the BBB disruption and increased infiltration of immune cells into the CNS [92,93]. For example, monocytes may infiltrate the mouse brain after 15 weeks of HFD, where a correlation between the number of monocyte-derived macrophages in the brain and body weight is observed [94]. Moreover, in a study, they examine the BBB disruption in the offspring of animals fed with HFD rich in PA, resulting in the offspring of HFD-fed mice showing an increase in the BBB disruption, probably due to a reduction in the cell projections of a special type of ependymal cells called tanycytes [95]. The tanycytic cell bodies are in the third ventricle with their projections reaching the brain parenchyma, contacting the arcuate nucleus (ARC) neurons and endothelial cells in this area [27], and they form a passive physical barrier that prevents molecules in the median eminence from diffusing dorsally into the ARC [96].

Obesity and chronic HFD have been associated with reduced cognitive function in animals and humans [28,97,98,99,100,101,102]. For instance, a study in mice showed that HFD feeding, which contained approximately 40–45% fat composed mainly of saturated fatty acids, triggered neuronal apoptosis and reduced the synaptic inputs in the ARC and the lateral hypothalamus [28,103]. Mice infused with PA have developed impaired synaptic plasticity, and their memory was affected by activated microglia and the release of TNF-α [26]. Moreover, orexin/ataxin-3 mice (transgenic mouse neurodegeneration models) fed with HFD were submitted to a two-way active avoidance (TWAA) hippocampus-dependent memory task that involves learning and the utilization of stimuli in the spatial environment. This experiment showed a clear impairment in learning and an increase in the microglial activation biomarker Iba-1, CX3 chemokine receptor 1 (CX3CR1), and TNF-α [104,105,106]. This was probably because neuronal populations within the hippocampus have a particularly high metabolic demand, making them vulnerable to a variety of environmental and biological factors [28]. Similarly, Giles et al., (2016) showed a relationship between the plasma concentration of FA and two regions of the brain using a multivariate analysis. They found that long-term enriched diets of SAFA in mice lead to changes in the hippocampus and cerebral cortex lipidomes. Moreover, it has been found that PA levels increased in the cerebrospinal fluids (CSF) in humans with obesity, which was inversely correlated with cognitive performance [26]. Different studies have revealed that excess PA may trigger different cellular pathways that could be implicated in cytotoxicity and cell death [107].

## 4. Pathways Involved in Palmitic Acid-Induced Toxicity

Some studies have associated SAFAs as a primary cause of cell death [108]. In this regard, it has been reported that PA could affect the cell viability of different brain cells, such as the neuroblastoma SH-SY5Y [109], glioblastoma T98G [110,111], microglial cells BV-2 [112], primary microglia [40], primary neurons [31], and primary astrocytes [113,114,115,116]. Although it is known that the PA could affect different brain cells through different cellular mechanisms, these are not clear. We will further describe the mechanisms to understand more about what underlies the damage caused by PA in the different brain cells that have been described to date (Table 2). This will be carried out to generate a better understanding of the effects of PA in the brain and the possible implications that excess PA produce in the brain, keeping in mind that this review also suggests the use of some other FAs to ameliorate the damages caused by PA dysregulation in the brain.

### 4.1. Role of Ceramides in Lipotoxic States

The ceramide is the precursor of all sphingolipids complex. It is abundant on neural cellular membranes and represents a potent regulator of brain homeostasis. However, when these lipids accumulate above a critical level, metabolism energy results are impaired. Intracellular levels of ceramide are fine-tuned, and alteration of the sphingolipid–ceramide profile contributes to the development of age-related, neurological, and neuroinflammatory diseases [133]. Several studies raise the possibility that ceramide accumulation in the hypothalamus and the increase of this lipid causes an upregulation in the expression and activity of serine palmitoyltransferase (SPT), neutral sphingomyelinase (n-mSMase), and the expression increase of NF-kB through ER stress or downstream effects [134,135,136]. Ceramides within the CNS may modulate cell damage with pro-inflammatory kinases, such as p38 mitogen-activated protein kinase and ceramide-activated protein (CAP) [108,137]. In PA-treated astrocytes, it has been reported that experimental downregulation of SPT decreases ceramide levels, mitigating the production of IL-1β and TNF-α secreted by the astrocytes [32]. As well as the direct effects on astrocytes, TLR2/4 factors activate microglia to increase inducible nitric oxide synthase (iNOS) and superoxide, leading to peroxynitrite that stimulates astrocyte sphingomyelinase (aSMase), leading to the production of longer chain ceramides, which, at sufficient levels, can be pro-apoptotic. Moreover, changes in astrocytes by ceramides have negative consequences on BBB integrity and affect oligodendrocytes’ functions [138]. Another detrimental effect of ceramide in the brain is that microglia increase the assembly of inflammasome that contains NOD, LRR, and pyrin domains, increasing the production of pro-inflammatory cytokines IL-1β and IL-18 [139]. On the other hand, researchers treated immortalized mouse Schwann cells (IMS) and rat primary Schwann cells with 500 µM PA, a ceramide analog (C2-ceramide), and inhibitors of de novo ceramide synthesis (myriocin and fumonisin B1). Myriocin and fumonisin B1 significantly attenuated the apoptosis of IMS cells caused by incubation with palmitate for 48 h, suggesting that ceramide could be the cause of apoptosis [140].

In most reports, it has been shown that increases in ceramide levels generate ROS, establishing a link between OS and sphingolipid metabolism with detrimental consequences in the brain. In primary hippocampal cultures, ROS levels were also increased in ceramide-treated cells in a dose-dependent manner [141]. In this regard, Darios et al., (2003) evaluated ceramide-mediated cell death in PC12 cells and found increased ROS production, a transient increase in cytosolic free calcium, and a long-lasting increase in mitochondrial free calcium. Interestingly, mitochondrial calcium did not increase because cytosolic free calcium levels rose [142].

### 4.2. Inflammation Pathways (TLR, NF-κB, and Cytokines)

In the brain, the inflammatory processes could be beneficial or detrimental, depending on the strength and duration of the response. Likewise, very strong and chronic activation of glial cells drives pro-inflammatory cytokine production, which could be related to NDs [23]. Some studies have suggested that CNS-expressed TLR4 may mediate the hypothalamic inflammation induced by HFD [143,144,145,146]. Furthermore, Korbecki and Bajdak-Rusinek (2019) described that TLR4 or TLR2 activation led to the activation of NF-kB that produced the activation of NLRP3-inflammasome, inducing pro-inflammatory signals and the recruitment to lipid rafts. Then, adding this to ROS generation will activate caspase 1, triggering inflammasome assembly and the maturation of pro-IL-1β to IL-1β [136]. Similarly, Jang et al., (2016) demonstrated that NLRP3 inflammasome is involved in the progression of NDs [147].

It has been shown that neurons can sense circulating free FAs and generate adaptive responses to high free FA levels through the expression of TLRs, such as TLR4 and TLR2 [148]. Besides, in mHypoA-POMC/GFP-2 neurons, PA activates NF-κB through the MAP kinases JNK and ERK [149], which is related to what was reported by Kwon et al., (2014), showing that cortical neurons treated with 300 µM PA for 24 h have higher levels of IL-6 expression [150]. Moreover, an increase in IL-6 expression was seen in primary hypothalamic neurons treated with 200 µM PA for 18 h [151]. However, Sergi et al., (2020) reported that the mHypoE-N42 hypothalamic neuron cell line, treated with 200 µM PA for 24 h, showed an upregulation IL-6 and TNF-α, but when the cells were treated with a TLR4 activator there was no change in the expression of IL-6 and TNF-α, suggesting that this upregulation is due to ceramide accumulation rather than TLR4 [43].

On the other hand, a study evaluated the effects of excess PA on astrocytes, showing that TLR4-dependent IL-6 and TNF-α release was independent of the presence of microglia to generate the response [91], suggesting this as a differential response from glia and neurons. Additionally, Chen et al., (2018) showed that hippocampal astrocytes treated with 250 µM PA for 4, 8, and 12 h induced inflammation and apoptosis that was regulated by the autophagic degradation of caveolin-1 [152]. Similarly, another study reported that the astrocytes of mice submitted to an HFD of 60% fat showed that their astrocytes activated their inflammatory response through the inhibition of the inhibitor of nuclear factor kappa-B kinase subunit beta (IkkB)/NF-κB [139]. Moreover, it was shown that PA activates IPAF inflammasome in primary astrocytes to release IL-1β, which damages primary neurons [118]. The previous evidence confirms that astrocytes treated with high amounts of PA will generate a strong inflammatory response that can affect neurons.

Like astrocytes, when microglia cells are treated with PA for 12 h, the expression of the subunit p65 of NF-κB increases approximately 60% compared to their control [112]. Likewise, another study reported that PA treatment on microglia increases the expression of interleukin-1 beta (IL-1b) and inducible nitric oxide synthase (iNOS). Then, PA induced the TLR4-mediated activation of NF-κB, which is responsible for TNF-α, IL-1b, and nitric oxide (NO) production [109]. This was confirmed with the results obtained by Tu et al., (2019), finding that the same mechanisms were induced, plus the expression of IL-6. Moreover, they validated their results with an animal model by obtaining similar results to the in vitro studies [40].

### 4.3. Lipotoxic Oxidative Stress

ROS can have different functions in the brain; it is known that ROS generated by microglia and astrocytes could modulate synaptic and non-synaptic communication between neurons and glia [153]. Moreover, different factors could generate an imbalance in the production and metabolism of ROS, leading to pathological conditions [153]. For instance, one of the main sources of OS is the activation of NADPH [154], which has been proven in the brain in different models, such as inflammation after ischemia [155], PD [156], and AD [157]. Notably, some studies show PA-induced activation of NADPH in different cells, such as the endothelial cells [158], hepatic cells [159], and even in neurons [130]. However, the direct link between PA and NADPH production has not been reported in other brain cells.

Different studies have demonstrated that a PA-rich HFD produces cellular damage in neurons, astrocytes, and microglia that is, in part, due to increased OS related to the overproduction of ROS, which can induce lipid droplet formation and accumulation, activating canonical inflammatory pathways and chronic inflammation [160,161]. Importantly, signs of OS precede the development of NDs by an impairment in hypothalamic genetic expression, as well as the downregulation of B-cell lymphoma-2 (Bcl-2) [162]. These OS effects given by PA appear in a cell-specific manner [160]. For example, Park et al., (2011) showed that neural progenitor cells (NPCs) are vulnerable to 200–400 μM PA, which causes cell death due to increased ROS and reduced NPC viability and proliferation. Furthermore, a short-term PA-rich HFD impairs hippocampal neurogenesis by decreasing the survival of newly generated cells and the expression levels of brain-derived neurotrophic factor (BDNF) [31]. Hsiao et al., (2014) showed PA-induced cytotoxicity in the neuroblastoma cell line SH-SY5Y-γ and the glioblastoma T98G cell line, in a time- and dose-dependent manner [109], revealing that PA induced apoptotic cell death in those cell lines, but the percentage of apoptosis was much lower in T98G with similar concentrations of PA treatment, suggesting that neurons are more susceptible to PA-induced cytotoxicity than astrocytes (Figure 1) and that PA-induced apoptotic cell death was associated with increased lipid peroxidation and ROS production.

Wong et al., (2014) showed that when cortical astrocytes from rats were submitted to 100 µM PA for 24 h, it triggered apoptotic cell death. However, in this study, PA-induced cell death appeared to be unrelated to ER stress and perturbation in cytosolic Ca^2+^ signaling. It was related to ROS production and a subsequent MMP collapse [117]. However, González-Giraldo et al., (2018) found that when the T98G cell line was treated with 1 mM PA for 24 h, it reduced cell viability by 50%; moreover, it reduced MMP by 56.1% and cardiolipin by 50%, but it did not generate an increase in ROS production, showing that PA treatment can induce a wide variety of responses and that, in this study, the damage was independent of ROS production [111]. Likewise, a study using PA on primary astrocytes showed that PA treatment for 24 h did not produce a significant increase in peroxide ions but increased the production of superoxide ions, which can be linked to the observed reduction of cardiolipin and the loss of MMP [121]. These results suggest that PA responses are heterogeneous, and it could be beneficial to not focus only on specific mechanisms as initial approaches but to explore a broader panorama using systems biology.

On the other hand, Hidalgo-Lanussa et al., (2018) reported that microglia subjected to PA damage for 12 h showed a reduction in viability, increased ROS production, and reduced mitochondrial mass, and MMP [112]. Importantly, similar results were found when studying the whole brain of rats, finding dysregulation on the primary antioxidant enzymes, such as superoxide dismutase (SOD), glutathione peroxidase (GPx), and catalase (CAT) [163]. Moreover, the antioxidative activity, such as SOD, CAT, and GPx, was measured in the hippocampus and the cerebral cortex of rats under HFD, augmenting the response of their activity. Nevertheless, this increase was not enough to prevent the damage caused by OS [164].

### 4.4. Endoplasmic Reticulum Stress Pathways

Disturbances of the ER have also been revealed in CNS injury. The pathological signals disturb protein post-translational modifications and disrupt homeostasis. This may result in the accumulation of unfolded or misfolded proteins in the ER lumen, a condition known as ER stress [165,166,167,168]. ER stress can be a result of high-caloric intake. This plays a key role in alterations in the metabolic regulation of pathologies, such as obesity, type 2 diabetes (T2D), prion diseases, PD, and AD [168,169,170].

PA-derived ER stress induces eukaryotic elongation factor (eEF) 1A-1, which affects the integrity of the cytoskeleton, causing cellular death [108]. A study using male Wistar rats fed an HFD showed that SAFAs activate ER stress in the hypothalamus [136,145]. Similar results were obtained in SH-SY5Y neurons treated with 1000 µM PA for 24 h, triggering ER stress, which was determined by the expression of spliced X-box binding protein-1 (XBP-1) mRNA and binding immunoglobulin protein (BiP). Furthermore, PA increased JNK activation and tau hyperphosphorylation and inactivated adenosine monophosphate (AMP)-activated protein kinase (AMPK) [171]. Hsiao et al., (2014) found that SH-SY5Y cells treated with 300 µM PA for 24 and 48 h had significantly arrested their cell cycle in the G2/M phase. This response was caused by ER stress, according to an increase in eukaryotic translation inhibition factor 2α, an ER stress marker [109]. In a similar study using SH-SY5Y and mouse Neuro-2a (N2a) neuroblastoma cells submitted to 100 µM palmitate for 24 h and an animal model of C57BL/6J mice fed an HFD with 2.20% *w*/*w* of PA, the PA caused ER stress in the mouse cortex and hippocampus in both cell lines. PA induced ER stress via CHOP expression and caused the inhibition of leptin and IGF1 expression at the transcriptional level. Their dysregulation is linked with the progression of different NDs [20]. This is related to the increase in CHOP generated by a PA treatment in mHypoA-POMC/GFP neurons. This response was mediated through the MAP kinases JNK and ERK, and the effects were dependent on palmitoyl-CoA synthesis [149]. On the other hand, Ortiz-Rodriguez et al., (2018) reported that cortical astrocytes from post-natal mouse males and females that were submitted to PA at 250 and 500 μM for 24 h showed an increase in the ER stress marker CHOP [115]. Interestingly, ER stress as a source of cell damage by PA has not been reported in the other brain cells.

### 4.5. Apoptosis Related to PA

In normal conditions, apoptosis, or programmed cell death, in the brain helps to regulate the development of the nervous system and the clearance of cells that are not working properly [172]. However, it is known that exposure to high levels of SAFAs, such as PA, triggers apoptosis in different brain cells [109,111,112,120,121]. It was shown that PC12 cells exposed to PA showed a reduction of cell viability after 24 h of treatment. The cell death induced by PA was apoptotic, which was confirmed by morphological analysis and the measurement of caspase-8 and caspase-3 activity. Moreover, western blotting showed that PC12 exhibited the signature apoptotic cleavage fragment of poly (ADP-ribose) polymerase (PARP) [173]. RT-PCR and RNA blot experiments showed an upregulation of the FA receptor and ligand mRNA, suggesting a role in apoptotic death [173]. Similarly, Yuan et al., (2013) reported that PA significantly impaired cell viability via apoptosis of neural stem cells (NSCs) in a dose- and time-dependent manner. Furthermore, they showed that there were increased protein levels of Bcl-2-associated X protein (Bax) and cleaved caspase 3, plus a reduced expression of Bcl-2 after PA treatment. Additionally, the expression of phospho-c-Jun N-terminal kinase (p-JNK) was increased significantly. Nevertheless, the JNK inhibitor effectively reduced the apoptosis of NSCs induced by PA [174]. It was shown that GL15 glioblastoma treated with PA generated a loss of cardiolipin that was related to apoptosis through the release of cytochrome c following the activation of caspase 3. Nevertheless, the cell apoptosis was not related to the ceramide pathway nor the mitochondrial pro-apoptotic AIF or Bcl-2/Bax factors [131]. Is important to highlight that although many studies report an increase in cell death after PA insult in brain cells, the exact mechanisms of death are not yet well established.

### 4.6. Autophagy and Palmitic Acid

Autophagy is responsible for the degradation of damaged proteins and organelles by surrounding the cytoplasmic components in the double-membrane vesicles called autophagosomes [132]. It is important to highlight that autophagy plays a crucial role in neuronal physiology, and the impairment of any step of the autophagic pathway usually generates axonal defects that culminate in neuronal degeneration [175]. It was demonstrated that the prolonged consumption of an HFD generates a blockade of autophagy in the hypothalamus [115,176]. Besides, the hypothalamic cell line N43/5 was treated with 100 µM PA for 24 h, activating the free fatty acid receptor 1 (FFAR1), also known as G protein-coupled receptor 40 (GPR40), demonstrating that exposure to PA inhibits autophagic flux and reduces insulin sensitivity [22]. Moreover, mice under an HFD for 8 or 16 weeks and in response to intracerebroventricular injections of PA showed that chronic exposure to an HFD leads to an increased expression of inflammatory markers and a downregulation of autophagic proteins. When autophagy was induced in obese mice, it was shown to reduce JNK and Bax expression and increase Bcl-2 activity [132].

Finally, Ortiz-Rodriguez et al., (2018) found that the effect of PA in reducing cell viability after 24 h was enhanced by the blockage of autophagic flux with hydroxychloroquine (an inhibitor of autophagosome-lysosome fusion). This suggests that autophagy impairment is involved in the effect of PA on astrocyte cell death [115]. It has been demonstrated that astrocytes can modulate autophagy as a response to prevent the formation of protein aggregates in neurons and to mediate mitochondrial repair from mitophagy [177,178]. On the other hand, PA reduces autophagy in astrocytes, favoring ROS production, and those factors make it more difficult for the recovery of a mitochondrial tubular structure [178].

## 5. Palmitic Acid and Neurogenerative Diseases

Neurodegenerative processes are characterized by morphological, anatomical, and functional changes giving, as a result, an early chronic neuronal loss. Moreover, NDs have different causes, such as inheritance, environment, or a mix of both [23]. NDs usually are characterized by the decline of cognitive functions, in most cases affecting memory and learning processes, as well as a progressive degeneration that results in the debilitating conditions of movement [179]. Some of these NDs are AD and other dementias, amyotrophic lateral sclerosis (ALS), PD, and Huntington’s disease, where AD is the most prevalent, accounting for 60–70% of dementia cases [23,179].

On the other hand, HFD is correlated with cognitive function decline [28,180]. It has been shown that in different models the PA may accumulate in the brain and generate inflammation processes [28]. In that sense, studies have shown that in obese people, the inflammation affects the arcuate nucleus of the hypothalamus, which regulates satiety and hunger, and generates a feeding loop [27,28,103]. Backing up this statement, Valdearcos et al., (2017) found that mice under a 42% HFD, rich in PA for four weeks, had increased levels of PA in the hypothalamus and that the enteral administration of palmitate-rich milk fat led to a rapid accumulation of PA in the arcuate nucleus [181]. In this order of ideas, increases in PA concentrations have been linked with different NDs [181]. Over the review, we have discussed several mechanisms involved in the progression of many NDs, where chronic inflammation, OS generated by the peroxidation of lipids, and PERK activation by ER stress are key pathological events of most of the disorders previously mentioned [136,138,182,183].

### 5.1. Alzheimer Disease

**Figure 3:**
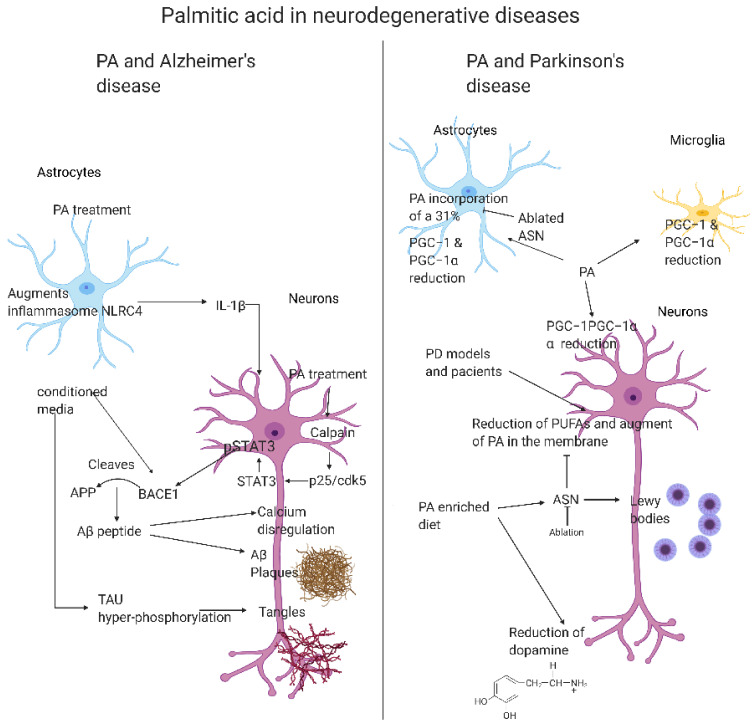
PA in neurodegenerative diseases. This figure shows the reported changes in brain cells induced by PA that are directly linked with the progression of Alzheimer’s and Parkinson’s diseases, as explained in Section 5. This figure shows how the relationship of astrocytes and microglia treated with PA will unleash a pro-inflammatory response that can induce the formation of hallmarks of AD, such as Aβ tangles and tau hyperphosphorylation, and of PD, causing the appearance of Lewy bodies, a reduction of dopamine, and ASN accumulation. Created with BioRender.com.

AD is the most common form of dementia and it is projected that in 2050 there will be 100 million patients with AD in the world [184]. The early symptoms of AD may include the loss of memory, apathy, and depression, while later symptoms include communication disorders, confusion, and behavioral changes, such as dysphagia [185]. Although, the causes of AD are not fully understood, there is a consensus of many genetic and environmental factors, including dietary FAs [20,34,186]. AD is associated with three conditions: neurofibrillary tangles or plaques of tau protein, the presence of amyloid β-protein (Aβ), and the proliferation of glial cells [186]. Abnormal tangles in the brain are formed with tau protein in the hyperphosphorylated form they are unable to bind to tubulin and microtubules, disrupting the neuronal cytoskeleton, as well as reducing microtubules and neurofilaments [187,188]. The plaques are composed of Aβ, and dysregulation of Aβ can generate insoluble aggregates of Aβ, which are toxic and are related to failures in cell-to-cell communication and neuronal death [34]. In addition, dietary FAs, such as PA, influence the inflammatory phenotype of glial and microglial cells [189]. As previously discussed, PA affects the NF-κB pathway, TLR-4 receptors, induces pro-inflammatory cytokines (IL-1β, IL-6, and TNF-α), and increases OS and ER stress, which are risk factors for AD [190,191]. Furthermore, these inflammatory responses lead to excessive glial activation that activates the kinases and drives the pathological phosphorylation of tau [186] (Figure 3).

Interestingly, Nasaruddin et al., (2016) reported an increase of 25% of the PA concentration in brains from people that had AD and an accumulation of 33% more in males compared with females [192]. Moreover, a study with rats fed a 60% HFD for 8 weeks, found that PA stimulates the expression of β-site amyloid precursor protein cleaving enzyme (BACE1) and amyloid precursor protein (APP) [193]. On the other hand, Bhattacharyya et al., (2013) and Zaręba-Kozioł et al., (2018), suggested that APP palmitoylation enhances Aβ peptide production through the amyloidogenic pathway and the dysregulation of palmitoylation in AD, due to Aβ oligomers being derived from the sequential proteolytic processing of APP using different enzymes, including BACE1, β-secretase, and γ-secretase [84,194]. APP is enriched in membrane lipid rafts forming dimers that induce the elevation of BACE1-mediated cleavage of the protein [84,194].

Cultures of primary cortical neurons treated with conditioned media from astrocytes submitted to 200 µM PA for 12 h showed that calcium levels were augmented by calpain activity, a calcium-dependent protease, which subsequently enhances p25/Cdk5 activity [195]. The authors showed that p25/Cdk5 uses STAT3 as a substrate. After phosphorylation it becomes active and is translocated into the nucleus. An elevated pSTAT3 level in the nucleus could transcriptionally upregulate both BACE1 and presenilin-1, enhancing the production of Aβ, and this could trigger neurons to disrupt calcium homeostasis, which is implicated in NDs, including AD [195]. Additionally, it has been shown that conditioned media of astrocytes submitted to PA induces AD-like hyperphosphorylation of tau in primary rat cortical neurons [29,196].

Patil et al., (2008) compared astrocytes from mouse brains from two different regions, the cortex (a region affected in AD) and the cerebellum (an unaffected region), which were treated with 200 µM PA. The conditioned medium was then transferred to the cortical neurons to study the possible effects on BACE1 upregulation and tau hyperphosphorylation [32]. It was found that the conditioned media from PA-treated cortical astrocytes but not the cerebellar astroglia increased the phosphorylation of tau and BACE1 expression [32,33]. Nevertheless, PA does not directly induce the AD-like changes in neurons, which could be explained by the low capability of neurons to take and metabolize FAs [32,96,118]. Similarly, PA activates the NLRC4 inflammasome in primary astrocytes to release IL-1β. When the levels of NLRC4 are reduced in the PA-treated astrocytes, it significantly diminishes IL-1β production. In addition, NLRC4 levels are upregulated in the brains of AD patients, suggesting a possible role of the NLRC4 inflammasome in AD pathogenesis [118].

### 5.2. Parkinson’s Disease

PD is a chronic and progressive ND that mainly affects the elderly population. Its symptoms appear gradually, and the early non-motor symptoms include hyposmia, fatigue, depression, behavioral disorders, and constipation [197,198]. The primary motor symptoms are bradykinesia, muscle stiffness, rigidity, and resting tremor. Later symptoms include postural instability, dysphagia, anxiety, orthostatic dizziness, urinary incontinence, sweating, and salivation [197,198]. The etiology of PD is influenced by genetic and environmental factors, and the symptoms of the disease are due to the degeneration of dopaminergic neurons [197,198]. Interestingly, Lewy bodies are observed in the areas of the brain affected by PD; it has been demonstrated that these bodies are primarily composed of ASN and form deposits in the cytoplasm of nerve cells [34]. Extensive research has accumulated evidence of the physiological function of ASN, based on its distribution in all major brain cell types [180,199,200]. ASN is related to the transport and control of neurotransmitter release, as well as modulating dopamine biosynthesis, the inflammatory response, the mobilization of synaptic vesicles in the nervous system, and the regulation of lipid and energy metabolism [199]. It has been found that only a small fraction of ASN (~4%) is phosphorylated in healthy brains and that a substantial accumulation of ASN phosphorylated with serine-129 (~90%) is observed in brains with Lewy pathology, showing an important association between this posttranslational modification and the accumulation of ASN aggregates [201].

On the other hand, several studies have approached the relationship between PA and PD [24,25,114,180,202]. In that sense, Shah et al., (2019) injected Sprague-Dawley rats unilaterally with 6-hydroxydopamine into the medial forebrain bundle to induce a loss of dopaminergic neurons in the substantia nigra, as a PD model. They found that PA was significantly increased in the PD model [180]. Likewise, Fabelo et al., (2011) used the human frontal cortex from people in the early motor stages of PD and incidental PD. They analyzed their lipid composition and found that the lipid rafts from PD and incidental PD cortices exhibit reductions in their contents of ω-3 and n-6 PUFAs. Importantly, the PA concentration was significantly higher than in control brains [24]. Schommer et al., (2018) found that a PA-enriched diet augmented ASN, which, combined with the depletion of dopaminergic neurons in the substantia nigra, are hallmarks of PD. Besides, they showed that the PA-enriched diet significantly reduced dopamine metabolites in m-Thy1 mice [25]. However, it was reported that mice with the ASN gene ablated reduced the incorporation of PA in the brain by 36% [198]. This was confirmed later by culturing astrocytes isolated from wild-type and ASN gene-ablated mice, showing a decrease in PA incorporation of 31% [203].

On the other side, recent evidence from Gonzalez-Riano et al., (2021), using a metabolomics approach to find PD biomarkers in the Spanish population, found a significant reduction of plasma PA in pre-PD subjects and they hypothesized that it was due to an early and progressive migration of PA from the plasma to the brain because ASN promoted the uptake of PA into the brain [202]. Besides, a reduced expression of peroxisome proliferator-activated receptor-gamma coactivator−1 α (PGC−1α) was found in affected brain tissue from PD patients, and it was found that PA stimulates PGC−1α promoter methylation in mouse primary cortical neurons, microglia, and astrocytes. PA caused PGC−1α gene promoter non-canonical cytosine methylation, reduced expression of the gene, and reduced mitochondrial content [204] (Figure 3).

### 5.3. Palmitic Acid and Other Neurodegenerative Disease

Multiple Sclerosis (MS) is an inflammatory, autoimmune, and chronic multifactorial disease of the CNS, and its symptoms are found in the sensory, cognitive, motor, and neuropsychiatric ranges [183,205]. It is caused by damage to the myelin sheath, leading to the malfunction of nerve impulses [206]. In patients with MS, it was reported that long SAFAs (>C16) were significantly lower, however, PA and palmitoleic acid were the only SAFAs that maintained a high concentration compared to the control [207]. Nevertheless, no significant differences were found in the analysis of the cerebrospinal fluid of patients with MS and control [208]. On the other hand, lipid peroxidation, which is a hallmark for many NDs, is one of the most important biomarkers in the pathogenesis of MS [183], where it has been reported that excessive levels of metabolites of PA, caused by lipid peroxidation, have been found in astrocytes and neurons [120,205]. Although, it would be interesting to study the direct effects of PA in MS or the lipidic profile in MS.

Huntington’s disease (HD) is an autosomal dominant ND resulting from the mutation in the huntingtin (HTT) gene that presents symptoms, such as cognitive, motor, and psychiatric signs [209]. The main characteristics are the reduction of striatal volume and the loss of medium spiny neurons of the striatum [210]. Huntingtin-interacting protein 14 (HIP14) is the most highly conserved of 23 human palmitoyl acyltransferases (PATs) that catalyze palmitoylation. It has been observed that HIP14 is dysfunctional in the presence of mutant HTT, and defective palmitoylation by HIP14 might be a relevant mechanism that contributes to the pathogenesis of HD [210]. Although there is not a direct link between PA treatment and HIP14, it could be an interesting topic for research because PA lipotoxicity alters palmitoylation in different models. Baldwin et al., (2012) suggested that PA-induced lipotoxicity is a consequence of uncontrolled protein palmitoylation, resulting in ER stress and apoptosis [211]. Besides, in PA-treated osteoblasts, PA induces negative changes in gene expression of palmitoyltransferase genes [212]. In SH-SY5Y cells, ER stress, cell cycle arrest, and cell death were attenuated by 2-bromopalmitate, a protein palmitoylation inhibitor, showing that palmitoylation plays an important role in the damage caused by PA-induced lipotoxicity [109]. Moreover, recent findings describe that GluN2B palmitoylation by acyl palmitoyl thioesterases (APTs) is reduced in the striatum in YAC128 mice and correlated with early degeneration in HD by the susceptibility of striatal neurons [213]. The above suggests that APTs could be agents that accelerate HD. This mechanism could be a potential treatment in early HD; however, further work is required [213].

## 6. The Therapeutic Potential of Other Fatty Acid in NDs

There is a growing need for searching for alternatives to treat neurodegenerative diseases because actual treatments have been proven ineffective or palliative [36]. Therefore, FAs have been investigated to assess if they have protective effects in the brain [38]. The PUFAs are a potential field of research, due to their relevant role in the CNS (neuroprotectants, fundamental in development, cell growth, and function). These PUFAs increase membrane fluidity and flexibility, as well as help microglia to increase phagocytosis [186]. Several studies suggested the influential role of PUFAs on the brain by the expression of phagocytic markers (CD206, Arg-1, and Ym-1) and anti-inflammatory cytokines (TGF-β and IL-10) by glial and microglial cells [38,41,186]. However, other FAs, such as oleic acid (OA) and linoleic acid (LA) exhibit neuroprotective effects in different models and could serve as potential therapeutic tools for NDs [40,43]. The experimental data is presented in Table 3.

### 6.1. Poly-Unsaturated Fatty Acids (PUFAs)

PUFAs serve as precursors of second messengers, such as the eicosanoids and docosanoids. For this reason, they are involved in the regulation of inflammatory response, immunity, blood vessels, platelets, synaptic plasticity, cellular growth, pain, sleep, and other processes [38]. Hence, many investigators have worked to determine the association of PUFAs with the protective pathways in the brain [41]. Moreover, experimental research of PUFAs using in vivo, and in vitro models suggests promising opportunities for developing therapies for AD, PD, HD, among other NDs [220]. For instance, Elharram et al., (2017) and Raefsky et al., (2018) demonstrated that deuterium-reinforced PUFAs (D-PUFA) are more resistant to lipid peroxidation mediated by ROS in comparison with regular hydrogenated PUFAs [221] and that in mouse models of cognitive impairment, with AD-like biochemical and structural pathologies, treatment with D-PUFA for 18 weeks showed a strong effect in the hippocampus and cortex. An approximate 55% reduction of F2-isoprostanes [222], which are biomarkers for OS in humans and are elevated in obesity [223], was observed. Moreover, prostaglandin F2α was reduced [217] and this compound is associated with neuropsychiatric and neurologic disorders [224]. Additionally, cells treated with MPP+ or 1-methyl-4-phenyl-1,2,3,6-tetrahydropyridine (MPTP) presented an increase in OS and reduced cell viability by ~70% and ~78.7%, respectively, compared to the control group. Nevertheless, both LA-, ARA-, and D-PUFA-supplemented mice indicate a neuroprotective role because PUFAs can inhibit MPP+ or MPTP in PC12 cells and may significantly slow oxidative cellular damage [220].

On the other hand, ω-3 PUFAs are well known for their importance in neuronal development and its neuroprotective function [225]. Indeed, it was reported that in mice ω-3 PUFAs also regulate CB1 activity, which is associated with signaling pathways in the prefrontal cortex and the nucleus accumbens [226,227]. DHA and eicosapentaenoic acid (EPA) are the most important long-chain fatty acids for cell membranes, myelin sheaths, oligodendrocytes, and nerve endings [225].

Additionally, protective effects exerted by PUFAs have been demonstrated in different studies, showing that intracerebroventricular administration of DHA decreases stroke volume and attenuates the induction of the pro-inflammatory signaling proteins NF-κB and COX2 [41,215]. Besides, augmented levels of DHA in the brain normalize BDNF levels in rats exposed to traumatic brain injury [228]. Consistent with this, the addition of DHA increased BDNF levels in astrocytes in vitro, and DHA deprivation decreased BDNF levels in the rodent brain [41,229]. Furthermore, there is evidence that 50 µM DHA ameliorates and even reverses the damages generated by PA-induced lipotoxicity in Schwann cells. This effect is mediated, at least in part, by the activation of the PI3K/AKT and mTORC2 pathways [42]. Moreover, neurons treated with TAG-DHA for neural viability, following the application of the neurotoxin 6-hydroxydopamine, showed reduced neuronal death, indicating that the use of TAG-DHA as a neuroprotective agent in PD may constitute a promising pharmacotherapeutic strategy [230].

There is evidence in different cohorts of the protective effects of ω-3 PUFAs in the brain. For instance, studies from organic milk demonstrated that it contains ~50% of ω-3 PUFAs, and that there exists a positive correlation between breastfeeding and cognitive development [38,231]. Likewise, Cooper et al., (2015) showed that supplementation with ω-3 PUFAs was significantly better for executive functioning and measures of IQ and processing speed by comparing the supplementation with EPA + DHA vs. placebo in a double-blind intervention [38,232]. Moreover, a 26-week randomized controlled trial gave (EPA 1.3 g + DHA 0.9 g per day) to 65 healthy older adults, showing that ω-3 PUFAs have beneficial effects on the brain and executive functions of cognition. Additionally, the treatment prevented a decrease in grey matter volume compared to the placebo group, and it had beneficial effects on the microstructural integrity of the white matter, carotid intima-media thickness, and diastolic blood pressure [233]. However, excessive intake of ω-3 PUFAs can have detrimental effects on the brain. For that reason, 3 g per day EPA + DHA is considered a safe and recommended dose [38].

PUFAs can protect from the neurodegenerative damage observed in different NDs, such as ALS. Fitzgerald et al., (2014) demonstrated that ω -3 PUFA intake was associated with a reduced risk of ALS, showing a correlation with both α-linolenic acid and marine n-3 PUFAs [234]. On the other side, brain slices of mice fed for 6 weeks with a diet that provides 0.8% EPA, and treated with MPP (+), showed that EPA treatment attenuated the MPP (+)-induced increase in ARA content in the brain and prevented the increase in the pro-apoptotic Bax and caspase-3 mRNAs [216]. Furthermore, the same group used the mouse model of PD (MPTP)-probenecid (MPTP-P) and EPA treatment, showing that EPA attenuated the MPTP-P-induced hypokinesia, ameliorated the procedural memory deficit, and suppressed the production of striatal pro-inflammatory cytokines. However, EPA did not prevent nigrostriatal dopamine loss [217]. After this, the same group evaluated in vitro the mechanisms in MPP (+)-treated cells. In this experiment, they showed that EPA attenuated the reduction of cell viability and prevented the presence of cytoplasmic inclusions. EPA treatment also exerted protective effects, such as ameliorating the MPP (+)-induced increase in tyrosine-related kinase B (TrkB) receptors, and downregulated ROS and nitric oxide, probably due to the inhibition of neuronal NADPH oxidase and COX-2. Similarly, EPA decreased the Bax: Bcl-2 ratio and cytochrome c release [218].

### 6.2. Linoleic Acid and Oleic Acid

There are other FAs with remarkable protective effects that could be used as a potential adjuvant to treat several deleterious effects present on many NDs. It has been observed that LA and alpha-LA (ALA) reduce the inflammatory response induced by PA treatment in the microglial cells of mice fed an HFD for 4 weeks [40]. Likewise, there is evidence that ALA and LA inhibit iNOS and COX-2 and ameliorated the cognitive dysfunction caused by Aβ and PA [235].

Based on the potential neuroprotective effects of LA and ALA, Piomelli (2013) suggested that OA ingestion stimulates oleoylethanolamide mobilization into the mucosal cells of the gut, which activates a PPARα-mediated signal that travels through the afferent vagus nerve to the hypothalamus, augmenting satiety, which has a beneficial effect for the system [236]. Besides, OA and EPA reduce the PA-induced intracellular ceramide accumulation, leading to a downregulation of IL-6 and TNF-α [43]. Indeed, co-administration of PA and OA improved performance in spatial learning and motor activity compared to the mouse that was fed only with PA [219]. Moreover, in mouse neuroblastoma Neuro-2a (N2a) cells submitted to an OA pre-treatment attenuated PA-induced mitochondrial dysfunction and insulin resistance by inhibiting the phosphorylation of mitogen-activated protein kinase and the nuclear translocation of NF-κB p65 induced by PA [150].

### 6.3. Medium and Short Chain Fatty Acids

There is evidence that medium-chain triglyceride ingestion improves cognition without adverse responses to hypoglycemia in intensively treated type 1 diabetic subjects [237]. These are a less harmful source for maintaining brain function because they are converted to medium-chain FAs (MCFAs) [237]. MCFAs may serve as agonists of peroxisome proliferator-activated receptors and are involved in several biological functions within the brain as enhancers of insulin sensitivity and acute inflammation. Moreover, they are involved in homeostatic functions, as well as energy processing, under pathological conditions (PD, MS, and epilepsy, among others) [238,239]. Importantly, the brain can generate ketone bodies through the oxidation of MCFAs [44]. Recent studies report the link between the use of ketone bodies and the improvement of AD and PD patients. Studies of animal models suggest that MCFAs can cross the BBB to be used by the brain and they do not require chylomicrons for transport or carnitine to enter the mitochondria [240]. Furthermore, MCFA supplementation produced therapeutic effects, such as the improvement of cognition in patients with AD and a reduction in neurodegeneration in a mouse model of ALS [44,241]. Additionally, MCFAs modulate astrocyte metabolism, such as in the case of decanoic acid, which induces metabolic changes, such as the acceleration of glycolysis, enhancing the astrocyte–neuron lactate shuttle, and octanoic acid, which does not affect lactate release but accelerates astrocyte ketogenesis [237]. Besides, the chain length of SAFAs in isocaloric diets affects insulin sensitivity, lipid metabolism, and mitochondrial fatty acid oxidation without influencing body weight. While dietary LCFA impairs insulin sensitivity and lipid metabolism, MCFA seems to protect from lipotoxicity and subsequent insulin resistance without caloric restriction [242].

On the other hand, short-chain FAs (SCFAs), which are molecules containing 2–5 carbon units, have shown benefits to the brain and are part of the link between “gut-health” and “brain-health” because they are naturally fermented by the gut flora, regulating appetite [44]. A study used treatments with SCFAs to ameliorate encephalomyelitis and reduce axonal damage via long-lasting imprinting on lamina-propria-derived gut T regulatory cells [243]. Nevertheless, more studies are needed to have a better understanding of their role in the brain and the gut–brain axis.

## 7. Conclusions and Further Perspectives

In this study, we have focused on PA and its role in causing many detrimental effects in the different types of brain cells. According to the literature, PA lipotoxicity involves ER stress, ROS production, an inflammatory response, and autophagy impairment. It is relevant to highlight that all the above factors can generate cellular damage and cell death. Excess PA has been linked to the principal hallmarks of different NDs such as Aβ peptide synthesis, tau hyperphosphorylation, calcium dysregulation, and ASN accumulation, among others. Interestingly, in the brain tissue of patients that developed different NDs, abnormally high concentrations of PA were found. These could suggest that PA is a potential causal factor behind cellular damage and NDs and a possible therapeutic target for multiple substances.

In that sense, there is growing evidence that other FAs can significantly ameliorate the lipotoxic effects of PA through diverse pathways. In this review, we focused on those FAs that exert neuroprotective functions and hold promise to treat the symptoms related to NDs. In addition, there is a lack of information linked to the effects of PA over NADH in the brain, very few studies using oligodendrocytes, and none applying 3D cell culture or organoids and PA. Therefore, further studies are necessary to continue elucidating the mechanism of PA’s effects in the brain and its relationship with neurodegenerative diseases. Importantly, the damages caused by PA in the different brain cells involves a wide diversity of mechanisms, activating different pathways in the same cells. Using holistic approaches, such as systems biology, could help to obtain better insights of the mechanisms working at a determined time. Finally, we found it necessary to continue exploring the potential effects of non-saturated fatty acids, SCFA, and MCFA as possible therapies for NDs.

## Figures and Tables

**Table 2:** List of the effects related to palmitic acid (PA) concentration and time of exposure to the insult in the different cells of the brain.

Reference	Species	Cell	Model	Concentration of PA (µM) and Time	Effects
Wong et al., 2014 [117]	R	Astrocytes Primary (P)	In vitro	100 (24 h)	Observations included ROS formation in the mitochondria, mitochondrial membrane potential (MMP) collapse, and apoptosis, excluding the involvement of ER stress in PA toxicity.
Ramirez et al., 2019 [116]	M	Astrocytes P	In vitro	100 (24 h)	PA activated the Nrf2 pathway, reducing SOD activity and cell viability.
Tu et al., 2019 [40]	M	Microglia BV-2 cells	In vitro-In vivo	200 (4 h); HFD 60% (4 w)	Increases were observed in IL-6, IL-1 β, TNF-α, and COX2 expression and the ratio of phospho extracellular signal-regulated kinases (pERK)/ERK and a reduction of IkBα an Nf-kB inhibitor was observed. The same effects were seen in an animal model, plus the activation of microglia.
Sergi et al., 2020 [43]	M	Neurons P	In vitro	200 (6 h and 24 h)	PA induced the expression of IL-6 and TNF-α independent of TLR4 but, partially, via ceramide synthesis.
Gupta et al., 2012 [91]	R	Astrocytes P	In vitro	200 (18 h)	Astrocytes liberated IL-6 and TNF-α via TLR4 but not c-Jun N-terminal kinase (JNK) and TLR2.
Hidalgo-Lanussa et al., 2018 [112]	M	Microglia BV-2 cells	In vitro	250 (12 h)	PA increased ROS production and Nf-kB expression and also reduced MMP, cardiolipin, and cell viability.
Liu and Chan, 2014 [118]	R	Astrocytes and neurons P	In vitro	400 (24 h)	Activation of IPAF-ASC inflammasome in astrocytes led to the maturation of IL-1β, and neurons treated with the conditioned media of astrocytes with PA increased amyloid β42.
Frago et al., 2017 [113]	R *	Astrocytes P	In vitro	500 (24 h)	PA reduced the activation of ERK and Akt and the expression of IL10 and aromatase. PA also, augmented the activation of P38 mitogen-activated protein kinases, JNK, and the expression of IL-6. (C/EBP homologous protein (CHOP) and caspase 3are related to endoplasmic reticulum stress.)
Gonzalez-Giraldo et al., 2019 [119]	H	Astrocytes t98 g cells	In vitro	1000 (24 h)	PA increased IL6, TERT, TERC, DNMT3B, ESR1, and MIR155 genes and reduced CREB1, ALDH1L1, IL1B, and MIR125a.
Gonzalez-Giraldo et al., 2018 [111]	H	Astrocytes t98 g cells	In vitro	1000 (24 h)	PA reduced MMP, cardiolipin, and cell viability
Yee-Wen et al., 2018 [120]	H	Astrocytes t98 g cells and Neurons SH-SY5Y	In vitro	100–500 (24 h and 48 h)	PA induced apoptotic cell death in the SH-SY5Y and T98G cell lines, and treatment with similar concentrations of PA showed a much lower percentage of apoptosis in the T98G line, indicating that neurons are more susceptible to PA. These results were associated with increased lipid peroxidation and ROS production.
Martin-Jimenez et al., 2020 [121]	H	Astrocytes, Normal Human Astrocytes primary	In vitro	2000 (24)	Astrocytes treated with PA showeda reduction in cardiolipin and MMP and an increase in superoxide production, nuclear fragmentation, and cell death.
Hsiao et al., 2014 [109]	H	Neurons SH-SY5Y	In vitro	100–500 (24 h and 48 h)	Neuronal cell apoptosis, cell cycle G2/M arrest, beta-amyloid accumulation and the elevation of endothelial reticulum stress were observed. All of these effects were reversed by inhibiting protein palmitoylation.
Ortiz-Rodriguez et al., 2018 [115]	M * pre-natal	Astrocytes P	In vitro	250–500 (24 h)	PA reduced LC3-II, an autophagy marker, and incremented expression of CHOP, IL-6, and, only in males, TNF-α. Increased cell death was observed.
Wang et al., 2012 [122]	M	Microglia P	In vitro	25–200 (6 h and 24 h)	PA increased the expression of IL-6 and the TLR4-mediated activation of NF-kB, which was responsible for increases in TNF-a, IL-1b, and NO production.
Yudkoff et al., 1989 [123]	R	Astrocytes P	In vitro	360–720 (24 h)	PA reduced intracellular glutamine concentrations and increased leucine, isoleucine, and taurine.
Park et al., 2011 [31]	M pre-natal	Neurons neural progenitor cells	In vitro	50, 100, 200, and 400 (24 h)	PA was found to reduce NPC viability and proliferation by elevating intracellular OS. Furthermore, short-term PA-rich HFD impaired hippocampal neurogenesis by reducing the survival of newly generated cells and BDNF levels in the hippocampus.
Ramirez et al., 2019 [116]	M	Astrocytes P	In vitro	200 (2 h, 6 h, and 24 h); HFD 50% (8 w)	PA augmented ROS production and reduced SOD expression. In addition, in the animal model a reduction of BDNF was seen.
Morselli et al., 2016 [124]	M *	Brain	In vivo	HFD 42% (16 w)	An HFD rich in SAFAs caused hypothalamic inflammation, principally in males, which was related to the decrease of PUFAs in the brain and the down regulation of PGC-1α/Erα.
Douglass et al., 2017 [125]	M	Brain	In vivo	HFD 60% (8 w)	An HFD rich in SAFAs augmented hypothalamic inflammation and astrocytosis through the activation of Nf-kB and IKKβ.
Blázquez et al., 2001 [126]	R	Astrocytes cortical	In vitro	200 (24 h, 48 h, and 72 h)	PA induced apoptosis and involved the de novo synthesis of ceramide through the Raf-1/ERK pathway.
Escartin et al., 2007 [127]	R	Astrocytes P	In vitro	200 (24 h)	CNTF induced resistance to palmitic acid damage in activated astrocytes by increasing beta-oxidation.
Patil et al., 2007 [32]	R	Astrocytes cortical	In vitro	200 (24 h)	PA reduced GLUT1 expression, glucose uptake, and lactate release.
Tracy et al., 2013 [128]	R	Microglia BV-2 cells	In vitro	125 (24 h)	PA induced the activation of microglia, augmenting the mRNA levels of the proinflammatory cytokines Ia1β and IL-6.
Yan et al., 2016 [129]	R	Retinal ganglional cells RGC-5	In vitro	100 (24 h)	Cell death due to ROS levels rose.
Calvo-Ochoa et al., 2017 [130]	R	differentiated human neuroblastoma cells (MSN)	In vitro	200 (24 h)	Inhibition of the insulin/PI3K/Akt pathway was observed.
Buratta et al., 2008 [131]	H	Glioblastoma GL15	In vitro	600 (36 h)	PA generated a loss of cardiolipin, which was related to apoptosis via the release of cytochrome c and activation of caspase 3.
Hernández-Cáceres et al., 2019 [22]	R	hypothalamic cell line N43/5	In vitro	100 (24 h)	PA activated (GPER40) and PA inhibited the autophagic flux and reduced insulin sensitivity.
Portovedo et al., 2015 [132]	R	Brain	In vivo	HFD 35% 8–16 w	Intracerebroventricular injections of PA and HFD increased the expression of inflammatory markers and the downregulation of autophagic proteins.

M: mouse; R: rat; H: human; *: both sexes (male and female) cells tested; h: hours; w: weeks.

**Table 3:** Fatty acids (FAs) with protective effects in the brain against palmitic acid (PA)-induced damage.

Reference	FA	FA Concentration and (Time)	Model	Cells	Disease Model	Effects
Tang et al., 2014 [214]	ARA	50 µM (24 h)	M	Neurons PC12	PD	A 50 µM concentration showed protection against MPP. However, 100 µM generated cytotoxicity.
Marcheselli et al., 2003 [215]	DHA	100–200 ng (24 h–48 h)	M	Brain	LP	DHA generated neuroprotection, inhibiting leukocyte infiltration, NF-κB, and cyclooxygenase-2. It also inhibited the signaling response to ischemia reperfusion.
Descorbeth et al., 2018 [42]	DHA	50 µM (48 h)	R	Schwann cells	LP	DHA reduced cell death generated by PA through the activation of the PI3K/AKT and mTORC2 kinase pathways.
Meng et al., 2010 [216]	EPA	0.8% (6 w)	M	Brain	PD	EPA reduced the pro-apoptotic Bax and caspase-3 mRNAs.
Luchtman et al., 2012 [217]	EPA	0.8% (6 w)	M	Brain	PD	EPA reduced memory deficit and the production of pro-inflammatory cytokines in the striatum.
Luchtman et al., 2013 [218]	EPA	50 µM (48 h)	H	neurons SH-SY5Y	PD	EPA downregulated ROS and nitric oxide. Besides, NADPH oxidase and COX-2 attenuated an increase in the Bax: Bcl-2 ratio, and cytochrome c release. However, EPA did not prevent a decrease in MMP.
Tu et al., 2019 [40]	LA	15–30 (1 h)	M	Microglia BV-12	LP	LA reduced the expression IL-6, IL-1 β, TNF-α, COX2, reduced the ratio of pERK/ERK, inhibited IkBα and NF-κB, and reduced the activation of microglia.
Moazedi et al., 2007 [219]	OA	10% (4 w)	M	Brain	LP	Spatial learning and motor activity were significantly increased in rats fed with OA (10%) for 4 weeks compared to PA.
Kwon et al., 2014 [150]	OA	300 µM (24 h)	H	Neuron N2a	LP	OA pre-treatment attenuated PA-induced mitochondrial dysfunction and insulin resistance by inhibiting the phosphorylation of mitogen-activated protein kinase and the nuclear translocation of NF-κB p65.
Sergi et al., 2020 [43]	OA	125 µM (6 h)	M	mHypoE-N42	LP	OA counteracted PA-induced intracellular ceramide accumulation, leading to a downregulation of IL-6 and TNF-α via ceramide synthesis, with OA and EPA being anti-inflammatory by decreasing PA-induced intracellular ceramide build-up.
Govindarajan et al., 2011 [45]	NaB	1.2 g/kg (6 w)	M	Brain	AD	Sodium butyrate (NaB) generated a recovery of memory function that was correlated with elevated hippocampal histone acetylation and increased the expression of genes implicated in associative learning.
Ryu et al., 2003 [46]	NaB	1–30 mM (24 h)	R	Neurons P	HD	NaB reduced neuron death induced by OS through the activation of Sp1.

ARA: arachidonic acid; DHA: Docosahexaenoic acid; EPA: eicosapentaenoic acid; LA: linoleic acid; OA: Oleic acid; NaB: sodium butyrate; M: mouse; R: rat; H: human.

## Data Availability

Not applicable.

## References

[1] Hamilton J.A., Hillard C.J., Spector A.A., Watkins P.A. (2007). Brain uptake and utilization of fatty acids, lipids and lipoproteins: Application to neurological disorders. J. Mol. Neurosci..

[2] Tracey T.J., Steyn F.J., Wolvetang E.J., Ngo S.T. (2018). Neuronal Lipid Metabolism: Multiple Pathways Driving Functional Outcomes in Health and Disease. Front. Mol. Neurosci..

[3] Barber C.N., Raben D.M. (2019). Lipid metabolism crosstalk in the brain: Glia and neurons. Front. Cell. Neurosci..

[4] Panov A., Orynbayeva Z., Vavilin V., Lyakhovich V. (2014). Fatty acids in energy metabolism of the central nervous system. Biomed. Res. Int..

[5] Mesa-Herrera F., Taoro-González L., Valdés-Baizabal C., Diaz M., Marín R. (2019). Lipid and Lipid Raft Alteration in Aging and Neurodegenerative Diseases: A Window for the Development of New Biomarkers. Int. J. Mol. Sci..

[6] Frere S.G., Chang-Ileto B., Di Paolo G. (2012). Role of phosphoinositides at the neuronal synapse. Subcell. Biochem..

[7] Nakada-Tsukui K., Watanabe N., Maehama T., Nozaki T. (2019). Phosphatidylinositol Kinases and Phosphatases in Entamoeba histolytica. Front. Cell. Infect. Microbiol..

[8] Katan M., Cockcroft S. (2020). Phosphatidylinositol(4,5)bisphosphate: Diverse functions at the plasma membrane. Essays Biochem..

[9] Cermenati G., Mitro N., Audano M., Melcangi R.C., Crestani M., De Fabiani E., Caruso D. (2015). Lipids in the nervous system: From biochemistry and molecular biology to patho-physiology. Biochim. Biophys. Acta-Mol. Cell Biol. Lipids.

[10] De Fabiani E. (2014). Lipids IN the brain: Crossing the “insurmountable” barrier for a fatty, happy life. Eur. J. Lipid Sci. Technol..

[11] Lingwood C.A. (2011). Glycosphingolipid functions. Cold Spring Harb. Perspect. Biol..

[12] Liu M., Kelley M.H., Herson P.S., Hurn P.D. (2010). Neuroprotection of sex steroids. Minerva Endocrinol..

[13] Song Y., Kenworthy A.K., Sanders C.R. (2014). Cholesterol as a co-solvent and a ligand for membrane proteins. Protein Sci..

[14] Hashimoto M., Hossain S., Waisundara S.H.E.-V. (2018). Fatty Acids: From Membrane Ingredients to Signaling Molecules.

[15] Carta G., Murru E., Banni S., Manca C. (2017). Palmitic Acid: Physiological Role, Metabolism and Nutritional Implications. Front. Physiol..

[16] Clamp A.G., Ladha S., Clark D.C., Grimble R.F., Lund E.K. (1997). The influence of dietary lipids on the composition and membrane fluidity of rat hepatocyte plasma membrane. Lipids.

[17] Engin A.B., Engin A.B., Engin A. (2017). What Is Lipotoxicity? BT-Obesity and Lipotoxicity.

[18] Sorensen T.I.A., Virtue S., Vidal-Puig A. (2010). Obesity as a clinical and public health problem: Is there a need for a new definition based on lipotoxicity effects?. Biochim. Biophys. Acta-Mol. Cell Biol. Lipids.

[19] Unger R.H., Clark G.O., Scherer P.E., Orci L. (2010). Lipid homeostasis, lipotoxicity and the metabolic syndrome. Biochim. Biophys. Acta-Mol. Cell Biol. Lipids.

[20] Marwarha G., Claycombe K., Schommer J., Collins D., Ghribi O. (2016). Palmitate-induced Endoplasmic Reticulum stress and subsequent C/EBPα Homologous Protein activation attenuates leptin and Insulin-like growth factor 1 expression in the brain. Cell. Signal..

[21] Naumenko V.S., Ponimaskin E. (2018). Palmitoylation as a functional regulator of neurotransmitter receptors. Neural Plast..

[22] Hernández-Cáceres M.P., Toledo-Valenzuela L., Díaz-Castro F., Ávalos Y., Burgos P., Narro C., Peña-Oyarzun D., Espinoza-Caicedo J., Cifuentes-Araneda F., Navarro-Aguad F. (2019). Palmitic Acid Reduces the Autophagic Flux and Insulin Sensitivity through the Activation of the Free Fatty Acid Receptor 1 (FFAR1) in the Hypothalamic Neuronal Cell Line N43/5. Front. Endocrinol..

[23] Sochocka M., Diniz B.S., Leszek J. (2017). Inflammatory Response in the CNS: Friend or Foe?. Mol. Neurobiol..

[24] Fabelo N., Martín V., Santpere G., Marín R., Torrent L., Ferrer I., Díaz M. (2011). Severe alterations in lipid composition of frontal cortex lipid rafts from Parkinson’s disease and incidental Parkinson’s disease. Mol. Med..

[25] Schommer J., Marwarha G., Nagamoto-Combs K., Ghribi O. (2018). Palmitic Acid-Enriched Diet Increases α-Synuclein and Tyrosine Hydroxylase Expression Levels in the Mouse Brain. Front. Neurosci..

[26] Melo H.M., Seixas da Silva G.d.S., Sant’Ana M.R., Teixeira C.V.L., Clarke J.R., Miya Coreixas V.S., de Melo B.C., Fortuna J.T.S., Forny-Germano L., Ledo J.H. (2020). Palmitate Is Increased in the Cerebrospinal Fluid of Humans with Obesity and Induces Memory Impairment in Mice via Pro-inflammatory TNF-α. Cell Rep..

[27] Kim D.W., Glendining K.A., Grattan D.R., Jasoni C.L. (2016). Maternal Obesity in the Mouse Compromises the Blood-Brain Barrier in the Arcuate Nucleus of Offspring. Endocrinology.

[28] Rhea E.M., Salameh T.S., Logsdon A.F., Hanson A.J., Erickson M.A., Banks W.A. (2017). Blood-Brain Barriers in Obesity. AAPS J..

[29] Patil S., Chan C. (2005). Palmitic and stearic fatty acids induce Alzheimer-like hyperphosphorylation of tau in primary rat cortical neurons. Neurosci. Lett..

[30] Freitas H.R., Ferreira G.D., Trevenzoli I.H., Oliveira K.D., De Melo Reis R.A. (2017). Fatty Acids, Antioxidants and Physical Activity in Brain Aging. Nutrients.

[31] Park H.R., Kim J.-Y., Park K.-Y., Lee J. (2011). Lipotoxicity of Palmitic Acid on Neural Progenitor Cells and Hippocampal Neurogenesis. Toxicol. Res..

[32] Patil S., Melrose J., Chan C. (2007). Involvement of astroglial ceramide in palmitic acid-induced Alzheimer-like changes in primary neurons. Eur. J. Neurosci..

[33] Patil S., Balu D., Melrose J., Chan C. (2008). Brain region-specificity of palmitic acid-induced abnormalities associated with Alzheimer’s disease. BMC Res. Notes.

[34] Brodowicz J., Przegaliński E., Müller C.P., Filip M. (2018). Ceramide and Its Related Neurochemical Networks as Targets for Some Brain Disorder Therapies. Neurotox. Res..

[35] Coyne J.A. (2006). Comment on “Gene regulatory networks and the evolution of animal body plans”. Science.

[36] Durães F., Pinto M., Sousa E. (2018). Old Drugs as New Treatments for Neurodegenerative Diseases. Pharmaceuticals.

[37] Poovaiah N., Davoudi Z., Peng H., Schlichtmann B., Mallapragada S., Narasimhan B., Wang Q. (2018). Treatment of neurodegenerative disorders through the blood-brain barrier using nanocarriers. Nanoscale.

[38] Bentsen H. (2017). Dietary polyunsaturated fatty acids, brain function and mental health. Microb. Ecol. Health Dis..

[39] Kwon Y.-H., Kim J., Kim C.-S., Tu T.H., Kim M.-S., Suk K., Kim D.H., Lee B.J., Choi H.-S., Park T. (2017). Hypothalamic lipid-laden astrocytes induce microglia migration and activation. FEBS Lett..

[40] Tu T.H., Kim H., Yang S., Kim J.K.G., Kim J.K.G. (2019). Linoleic acid rescues microglia inflammation triggered by saturated fatty acid. Biochem. Biophys. Res. Commun..

[41] Bazinet R.P., Layé S. (2014). Polyunsaturated fatty acids and their metabolites in brain function and disease. Nat. Rev. Neurosci..

[42] Descorbeth M., Figueroa K., Serrano-Illán M., De León M. (2018). Protective effect of docosahexaenoic acid on lipotoxicity-mediated cell death in Schwann cells: Implication of PI3K/AKT and mTORC2 pathways. Brain Behav..

[43] Sergi D., Morris A.C., Kahn D.E., McLean F.H., Hay E.A., Kubitz P., MacKenzie A., Martinoli M.G., Drew J.E., Williams L.M. (2020). Palmitic acid triggers inflammatory responses in N42 cultured hypothalamic cells partially via ceramide synthesis but not via TLR4. Nutr. Neurosci..

[44] Romano A., Koczwara J.B., Gallelli C.A., Vergara D., Micioni Di Bonaventura M.V., Gaetani S., Giudetti A.M. (2017). Fats for thoughts: An update on brain fatty acid metabolism. Int. J. Biochem. Cell Biol..

[45] Govindarajan N., Agis-Balboa R.C., Walter J., Sananbenesi F., Fischer A. (2011). Sodium butyrate improves memory function in an alzheimer’s disease mouse model when administered at an advanced stage of disease progression. J. Alzheimers Dis..

[46] Ryu H., Lee J., Olofsson B.A., Mwidau A., Dedeoglu A., Escudero M., Flemington E., Azizkhan-Clifford J., Ferrante R.J., Ratan R.R. (2003). Histone deacetylase inhibitors prevent oxidative neuronal death independent of expanded polyglutamine repeats via an Sp1-dependent pathway. Proc. Natl. Acad. Sci. USA.

[47] Ebert D., Haller R.G., Walton M.E. (2003). Energy Contribution of Octanoate to Intact Rat Brain Metabolism Measured by ^13^C Nuclear Magnetic Resonance Spectroscopy. J. Neurosci..

[48] Schönfeld P., Reiser G. (2013). Why does Brain Metabolism not Favor Burning of Fatty Acids to Provide Energy?-Reflections on Disadvantages of the Use of Free Fatty Acids as Fuel for Brain. J. Cereb. Blood Flow Metab..

[49] Hall M.G., Quignodon L., Desvergne B. (2008). Peroxisome proliferator-activated receptor β/δ in the brain: Facts and hypothesis. PPAR Res..

[50] Huang Y., Thathiah A. (2015). Regulation of neuronal communication by G protein-coupled receptors. FEBS Lett..

[51] Bathina S., Das U.N. (2018). Dysregulation of PI3K-Akt-mTOR pathway in brain of streptozotocin-induced type 2 diabetes mellitus in Wistar rats. Lipids Health Dis..

[52] Callender J.A., Newton A.C. (2017). Conventional protein kinase C in the brain: 40 years later. Neuronal Signal..

[53] Kaltschmidt B., Kaltschmidt C. (2015). NF-KappaB in Long-Term Memory and Structural Plasticity in the Adult Mammalian Brain. Front. Mol. Neurosci..

[54] Sánchez-Alegría K., Flores-León M., Avila-Muñoz E., Rodríguez-Corona N., Arias C. (2018). PI3K Signaling in Neurons: A Central Node for the Control of Multiple Functions. Int. J. Mol. Sci..

[55] Weijers R.N.M. (2016). Membrane flexibility, free fatty acids, and the onset of vascular and neurological lesions in type 2 diabetes. J. Diabetes Metab. Disord..

[56] Moore S.A. (2001). Polyunsaturated fatty acid synthesis and release by brain-derived cells in vitro. J. Mol. Neurosci..

[57] Serhan C.N. (2014). Novel Pro-Resolving Lipid Mediators in Inflammation Are Leads for Resolution Physiology. Nature.

[58] Davletov B., Connell E., Darios F. (2007). Regulation of SNARE fusion machinery by fatty acids. Cell. Mol. Life Sci..

[59] Calon F., Lim G.P., Morihara T., Yang F., Ubeda O., Salem N., Frautschy S.A., Cole G.M. (2005). Dietary n-3 polyunsaturated fatty acid depletion activates caspases and decreases NMDA receptors in the brain of a transgenic mouse model of Alzheimer’s disease. Eur. J. Neurosci..

[60] Calon F., Lim G.P., Yang F., Morihara T., Teter B., Ubeda O., Rostaing P., Triller A., Salem N., Ashe K.H. (2004). Docosahexaenoic Acid Protects from Dendritic Pathology in an Alzheimer’s Disease Mouse Model. Neuron.

[61] Hansen H.S. (2013). Effect of Diet on Tissue Levels of Palmitoylethanolamide. CNS Neurol. Disord.-Drug Targets.

[62] Tsuboi K., Ikematsu N., Uyama T., Deutsch D.G., Ueda A.T. (2013). and N. Biosynthetic Pathways of Bioactive N-Acylethanolamines in Brain. CNS Neurol. Disord.-Drug Targets.

[63] Iannotti F.A., Di Marzo V., Petrosino S. (2016). Endocannabinoids and endocannabinoid-related mediators: Targets, metabolism and role in neurological disorders. Prog. Lipid Res..

[64] Skaper S.D., Facci L., Barbierato M., Zusso M., Bruschetta G., Impellizzeri D., Cuzzocrea S., Giusti P. (2015). N-Palmitoylethanolamine and Neuroinflammation: A Novel Therapeutic Strategy of Resolution. Mol. Neurobiol..

[65] Sanders S.S., Martin D.D.O., Butland S.L., Lavallée-Adam M., Calzolari D., Kay C., Yates III J.R., Hayden M.R. (2015). Curation of the Mammalian Palmitoylome Indicates a Pivotal Role for Palmitoylation in Diseases and Disorders of the Nervous System and Cancers. PLoS Comput. Biol..

[66] Blaskovic S., Blanc M., Goot F.G. (2013). What does S-palmitoylation do to membrane proteins?. FEBS J..

[67] Antinone S.E., Ghadge G.D., Lam T.T., Wang L., Roos R.P., Green W.N. (2013). Palmitoylation of superoxide dismutase 1 (SOD1) is increased for familial amyotrophic lateral sclerosis-linked SOD1 mutants. J. Biol. Chem..

[68] Young F.B., Butland S.L., Sanders S.S., Sutton L.M., Hayden M.R. (2012). Putting proteins in their place: Palmitoylation in Huntington disease and other neuropsychiatric diseases. Prog. Neurobiol..

[69] Zamzow D.R., Elias V., Acosta V.A., Escobedo E., Magnusson K.R. (2019). Higher levels of protein palmitoylation in the frontal cortex across aging were associated with reference memory and executive function declines. eNeuro.

[70] Stoeck A., Shang L., Dempsey P.J. (2010). Sequential and gamma-secretase-dependent processing of the betacellulin precursor generates a palmitoylated intracellular-domain fragment that inhibits cell growth. J. Cell Sci..

[71] Lievens P.M.-J., Kuznetsova T., Kochlamazashvili G., Cesca F., Gorinski N., Galil D.A., Cherkas V., Ronkina N., Lafera J., Gaestel M. (2016). ZDHHC3 Tyrosine Phosphorylation Regulates Neural Cell Adhesion Molecule Palmitoylation. Mol. Cell. Biol..

[72] Thorne R.F., Ralston K.J., de Bock C.E., Mhaidat N.M., Zhang X.D., Boyd A.W., Burns G.F. (2010). Palmitoylation of CD36/FAT regulates the rate of its post-transcriptional processing in the endoplasmic reticulum. Biochim. Biophys. Acta-Mol. Cell Res..

[73] Rothstein J.D. (2009). Current hypotheses for the underlying biology of amyotrophic lateral sclerosis. Ann. Neurol..

[74] Liu R., Wang D., Shi Q., Fu Q., Hizon S., Xiang Y.K. (2012). Palmitoylation Regulates Intracellular Trafficking of β2 Adrenergic Receptor/Arrestin/Phosphodiesterase 4D Complexes in Cardiomyocytes. PLoS ONE.

[75] Lu W., Roche K.W. (2012). Posttranslational regulation of AMPA receptor trafficking and function. Curr. Opin. Neurobiol..

[76] Oddi S., Dainese E., Sandiford S., Fezza F., Lanuti M., Chiurchiù V., Totaro A., Catanzaro G., Barcaroli D., De Laurenzi V. (2011). Effects of palmitoylation of Cys415 in helix 8 of the CB1 cannabinoid receptor on membrane localization and signalling. Br. J. Pharmacol..

[77] Yang X., Guo Z., Sun F., Li W., Alfano A., Shimelis H., Chen M., Brodie A.M.H., Chen H., Xiao Z. (2011). Novel membrane-associated androgen receptor splice variant potentiates proliferative and survival responses in prostate cancer cells. J. Biol. Chem..

[78] Zheng H., Pearsall E.A., Hurst D.P., Zhang Y., Chu J., Zhou Y., Reggio P.H., Loh H.H., Law P.-Y. (2012). Palmitoylation and membrane cholesterol stabilize μ-opioid receptor homodimerization and G protein coupling. BMC Cell Biol..

[79] Lakkaraju A.K.K., Abrami L., Lemmin T., Blaskovic S., Kunz B., Kihara A., Dal Peraro M., van der Goot F.G. (2012). Palmitoylated calnexin is a key component of the ribosome–translocon complex. EMBO J..

[80] Henderson M.X., Wirak G.S., Zhang Y.-q., Dai F., Ginsberg S.D., Dolzhanskaya N., Staropoli J.F., Nijssen P.C.G., Lam T.K.T., Roth A.F. (2016). Neuronal ceroid lipofuscinosis with DNAJC5/CSPα mutation has PPT1 pathology and exhibit aberrant protein palmitoylation. Acta Neuropathol..

[81] Gonzalo S., Linder M.E. (1998). SNAP-25 palmitoylation and plasma membrane targeting require a functional secretory pathway. Mol. Biol. Cell.

[82] Greaves J., Chamberlain L.H. (2007). Palmitoylation-dependent protein sorting. J. Cell Biol..

[83] Sohn H., Park M. (2019). Palmitoylation-mediated synaptic regulation of AMPA receptor trafficking and function. Arch. Pharm. Res..

[84] Zaręba-Kozioł M., Figiel I., Bartkowiak-Kaczmarek A., Włodarczyk J. (2018). Insights into Protein S-Palmitoylation in Synaptic Plasticity and Neurological Disorders: Potential and Limitations of Methods for Detection and Analysis. Front. Mol. Neurosci..

[85] Levental I., Grzybek M., Simons K. (2010). Greasing Their Way: Lipid Modifications Determine Protein Association with Membrane Rafts. Biochemistry.

[86] Henis Y.I., Hancock J.F., Prior I.A. (2009). Ras acylation, compartmentalization and signaling nanoclusters (Review). Mol. Membr. Biol..

[87] Giles C., Takechi R., Mellett N.A., Meikle P.J., Dhaliwal S., Mamo J.C. (2016). The Effects of Long-Term Saturated Fat Enriched Diets on the Brain Lipidome. PLoS ONE.

[88] Engin A., Engin A.B., Engin A. (2017). Eat and Death: Chronic Over-Eating BT-Obesity and Lipotoxicity.

[89] Virtue S., Vidal-Puig A. (2010). Adipose tissue expandability, lipotoxicity and the Metabolic Syndrome—An allostatic perspective. Biochim. Biophys. Acta-Mol. Cell Biol. Lipids.

[90] Karmi A., Iozzo P., Viljanen A., Hirvonen J., Fielding B.A., Virtanen K., Oikonen V., Kemppainen J., Viljanen T., Guiducci L. (2010). Increased brain fatty acid uptake in metabolic syndrome. Diabetes.

[91] Gupta S., Knight A.G., Gupta S., Keller J.N., Bruce-Keller A.J. (2012). Saturated long-chain fatty acids activate inflammatory signaling in astrocytes. J. Neurochem..

[92] Nerurkar P.V., Johns L.M., Buesa L.M., Kipyakwai G., Volper E., Sato R., Shah P., Feher D., Williams P.G., Nerurkar V.R. (2011). Momordica charantia (bitter melon) attenuates high-fat diet-associated oxidative stress and neuroinflammation. J. Neuroinflammation.

[93] Tucsek Z., Toth P., Sosnowska D., Gautam T., Mitschelen M., Koller A., Szalai G., Sonntag W.E., Ungvari Z., Csiszar A. (2014). Obesity in aging exacerbates blood-brain barrier disruption, neuroinflammation, and oxidative stress in the mouse hippocampus: Effects on expression of genes involved in beta-amyloid generation and Alzheimer’s disease. J. Gerontol. A Biol. Sci. Med. Sci..

[94] Buckman L.B., Thompson M.M., Lippert R.N., Blackwell T.S., Yull F.E., Ellacott K.L.J. (2014). Evidence for a novel functional role of astrocytes in the acute homeostatic response to high-fat diet intake in mice. Mol. Metab..

[95] Severi I., Fosca M., Colleluori G., Marini F., Imperatori L., Senzacqua M., Di Vincenzo A., Barbatelli G., Fiori F., Rau J.V. (2021). High-fat diet impairs mouse median eminence: A study by transmission and scanning electron microscopy coupled with raman spectroscopy. Int. J. Mol. Sci..

[96] Rodríguez E.M., Blázquez J.L., Pastor F.E., Peláez B., Peña P., Peruzzo B., Amat P. (2005). Hypothalamic Tanycytes: A Key Component of Brain–Endocrine Interaction. Int. Rev. Cytol..

[97] Cook R.L., O’Dwyer N.J., Donges C.E., Parker H.M., Cheng H.L., Steinbeck K.S., Cox E.P., Franklin J.L., Garg M.L., Rooney K.B. (2017). Relationship between Obesity and Cognitive Function in Young Women: The Food Mood and Mind Study. J. Obes..

[98] Farr S.A., Yamada K.A., Butterfield D.A., Abdul H.M., Xu L., Miller N.E., Banks W.A., Morley J.E. (2008). Obesity and Hypertriglyceridemia Produce Cognitive Impairment. Endocrinology.

[99] Goodman T., Hajihosseini M.K. (2015). Hypothalamic tanycytes-masters and servants of metabolic, neuroendocrine, and neurogenic functions. Front. Neurosci..

[100] Prickett C., Brennan L., Stolwyk R. (2015). Examining the relationship between obesity and cognitive function: A systematic literature review. Obes. Res. Clin. Pract..

[101] Tan B.L., Norhaizan M.E. (2019). Effect of high-fat diets on oxidative stress, cellular inflammatory response and cognitive function. Nutrients.

[102] Walker J.M., Dixit S., Saulsberry A.C., May J.M., Harrison F.E. (2017). Reversal of high fat diet-induced obesity improves glucose tolerance, inflammatory response, β-amyloid accumulation and cognitive decline in the APP/PSEN1 mouse model of Alzheimer’s disease. Neurobiol. Dis..

[103] Moraes J.C., Coope A., Morari J., Cintra D.E., Roman E.A., Pauli J.R., Romanatto T., Carvalheira J.B., Oliveira A.L.R., Saad M.J. (2009). High-fat diet induces apoptosis of hypothalamic neurons. PLoS ONE.

[104] Granholm A.-C., Bimonte-Nelson H.A., Moore A.B., Nelson M.E., Freeman L.R., Sambamurti K. (2008). Effects of a saturated fat and high cholesterol diet on memory and hippocampal morphology in the middle-aged rat. J. Alzheimers. Dis..

[105] Stranahan A.M., Norman E.D., Lee K., Cutler R.G., Telljohann R.S., Egan J.M., Mattson M.P. (2008). Diet-induced insulin resistance impairs hippocampal synaptic plasticity and cognition in middle-aged rats. Hippocampus.

[106] Duffy C.M., Hofmeister J.J., Nixon J.P., Butterick T.A. (2019). High fat diet increases cognitive decline and neuroinflammation in a model of orexin loss. Neurobiol. Learn. Mem..

[107] Chavez J.A., Holland W.L., Bär J., Sandhoff K., Summers S.A. (2005). Acid Ceramidase Overexpression Prevents the Inhibitory Effects of Saturated Fatty Acids on Insulin Signaling. J. Biol. Chem..

[108] Drosatos K., Schulze P.C. (2013). Cardiac Lipotoxicity: Molecular Pathways and Therapeutic Implications. Curr. Heart Fail. Rep..

[109] Hsiao Y.-H., Lin C.-I., Liao H., Chen Y.-H., Lin S.-H. (2014). Palmitic Acid-Induced Neuron Cell Cycle G2/M Arrest and Endoplasmic Reticular Stress through Protein Palmitoylation in SH-SY5Y Human Neuroblastoma Cells. Int. J. Mol. Sci..

[110] Chen X., Xu S., Wei S., Deng Y., Li Y., Yang F., Liu P. (2016). Comparative Proteomic Study of Fatty Acid-treated Myoblasts Reveals Role of Cox-2 in Palmitate-induced Insulin Resistance. Sci. Rep..

[111] González-Giraldo Y., Garcia-Segura L.M., Echeverria V., Barreto G.E. (2018). Tibolone Preserves Mitochondrial Functionality and Cell Morphology in Astrocytic Cells Treated with Palmitic Acid. Mol. Neurobiol..

[112] Hidalgo-Lanussa O., Ávila-Rodriguez M., Baez-Jurado E., Zamudio J., Echeverria V., Garcia-Segura L.M., Barreto G.E. (2018). Tibolone Reduces Oxidative Damage and Inflammation in Microglia Stimulated with Palmitic Acid through Mechanisms Involving Estrogen Receptor Beta. Mol. Neurobiol..

[113] Frago L.M., Canelles S., Freire-Regatillo A., Argente-Arizón P., Barrios V., Argente J., Garcia-Segura L.M., Chowen J.A. (2017). Estradiol Uses Different Mechanisms in Astrocytes from the Hippocampus of Male and Female Rats to Protect against Damage Induced by Palmitic Acid. Front. Mol. Neurosci..

[114] Martin-Jiménez C.A., Gaitán-Vaca D.M., Echeverria V., González J., Barreto G.E. (2017). Relationship Between Obesity, Alzheimer’s Disease, and Parkinson’s Disease: An Astrocentric View. Mol. Neurobiol..

[115] Ortiz-Rodriguez A., Acaz-Fonseca E., Boya P., Arevalo M.A., Garcia-Segura L.M. (2018). Lipotoxic Effects of Palmitic Acid on Astrocytes Are Associated with Autophagy Impairment. Mol. Neurobiol..

[116] Ramírez D., Saba J., Turati J., Carniglia L., Imsen M., Mohn C., Scimonelli T., Durand D., Caruso C., Lasaga M. (2019). NDP-MSH reduces oxidative damage induced by palmitic acid in primary astrocytes. J. Neuroendocrinol..

[117] Wong K.-L., Wu Y.-R., Cheng K.-S., Chan P., Cheung C.-W., Lu D.-Y., Su T.-H., Liu Z.-M., Leung Y.-M. (2014). Palmitic acid-induced lipotoxicity and protection by (+)-catechin in rat cortical astrocytes. Pharmacol. Rep..

[118] Liu L., Chan C. (2014). IPAF inflammasome is involved in interleukin-1β production from astrocytes, induced by palmitate; implications for Alzheimer’s Disease. Neurobiol. Aging.

[119] González-Giraldo Y., Forero D.A., Echeverria V., Garcia-Segura L.M., Barreto G.E. (2019). Tibolone attenuates inflammatory response by palmitic acid and preserves mitochondrial membrane potential in astrocytic cells through estrogen receptor beta. Mol. Cell. Endocrinol..

[120] Ng Y.W., Say Y.H. (2018). Palmitic acid induces neurotoxicity and gliatoxicity in SH-SY5Y human neuroblastoma and T98G human glioblastoma cells. PeerJ.

[121] Martin-Jiménez C., González J., Vesga D., Aristizabal A., Barreto G.E. (2020). Tibolone Ameliorates the Lipotoxic Effect of Palmitic Acid in Normal Human Astrocytes. Neurotox. Res..

[122] Wang Z., Liu D., Wang F., Liu S., Zhao S., Ling E.-A.A., Hao A. (2012). Saturated fatty acids activate microglia via Toll-like receptor 4/NF-κB signalling. Br. J. Nutr..

[123] Yudkoff M., Zaleska M.M., Nissim I., Nelson D., ErecinAska M. (1989). Neuronal Glutamine Utilization: Pathways of Nitrogen Transfer tudied with [15N]Glutamine. J. Neurochem..

[124] Morselli E., Frank A.P., Palmer B.F., Rodriguez-Navas C., Criollo A., Clegg D.J. (2015). A sexually dimorphic hypothalamic response to chronic high-fat diet consumption. Int. J. Obes..

[125] Douglass J.D., Dorfman M.D., Fasnacht R., Shaffer L.D., Thaler J.P. (2017). Astrocyte IKKβ/NF-κB signaling is required for diet-induced obesity and hypothalamic inflammation. Mol. Metab..

[126] Blázquez C., Geelen M.J., Velasco G., Guzmán M. (2001). The AMP-activated protein kinase prevents ceramide synthesis de novo and apoptosis in astrocytes. FEBS Lett..

[127] Escartin C., Pierre K., Colin A., Brouillet E., Delzescaux T., Guillermier M., Dhenain M., Déglon N., Hantraye P., Pellerin L. (2007). Activation of Astrocytes by CNTF Induces Metabolic Plasticity and Increases Resistance to Metabolic Insults. J. Neurosci..

[128] Tracy L.M., Bergqvist F., Ivanova E.V., Jacobsen K.T., Iverfeldt K. (2013). Exposure to the saturated free fatty acid palmitate alters BV-2 microglia inflammatory response. J. Mol. Neurosci..

[129] Yan P., Tang S., Zhang H., Guo Y., Zeng Z., Wen Q. (2016). Nerve growth factor protects against palmitic acid-induced injury in retinal ganglion cells. Neural Regen. Res..

[130] Calvo-Ochoa E., Sánchez-Alegría K., Gómez-Inclán C., Ferrera P., Arias C. (2017). Palmitic acid stimulates energy metabolism and inhibits insulin/PI3K/AKT signaling in differentiated human neuroblastoma cells: The role of mTOR activation and mitochondrial ROS production. Neurochem. Int..

[131] Buratta M., Castigli E., Sciaccaluga M., Pellegrino R.M., Spinozzi F., Roberti R., Corazzi L. (2008). Loss of cardiolipin in palmitate-treated GL15 glioblastoma cells favors cytochrome c release from mitochondria leading to apoptosis. J. Neurochem..

[132] Portovedo M., Ignacio-Souza L.M., Bombassaro B., Coope A., Reginato A., Razolli D.S., Torsoni M.A., Torsoni A.S., Leal R.F., Velloso L.A. (2015). Saturated fatty acids modulate autophagy’s proteins in the hypothalamus. PLoS ONE.

[133] Mencarelli C., Martinez-Martinez P. (2013). Ceramide function in the brain: When a slight tilt is enough. Cell. Mol. Life Sci..

[134] Cruciani-Guglielmacci C., López M., Campana M., le Stunff H. (2017). Brain Ceramide Metabolism in the Control of Energy Balance. Front. Physiol..

[135] Kakazu E., Mauer A.S., Yin M., Malhi H. (2016). Hepatocytes release ceramide-enriched pro-inflammatory extracellular vesicles in an IRE1 α -dependent manner. J. Lipid Res..

[136] Korbecki J., Bajdak-Rusinek K. (2019). The effect of palmitic acid on inflammatory response in macrophages: An overview of molecular mechanisms. Inflamm. Res..

[137] Carobbio S., Rodriguez-Cuenca S., Vidal-Puig A. (2011). Origins of metabolic complications in obesity: Ectopic fat accumulation. The importance of the qualitative aspect of lipotoxicity. Curr. Opin. Clin. Nutr. Metab. Care.

[138] Anderson G., Rodriguez M., Reiter R.J. (2019). Multiple sclerosis: Melatonin, orexin, and ceramide interact with platelet activation coagulation factors and gut-microbiome-derived butyrate in the circadian dysregulation of mitochondria in glia and immune cells. Int. J. Mol. Sci..

[139] Scheiblich H., Schlütter A., Golenbock D.T., Latz E., Martinez-Martinez P., Heneka M.T. (2017). Activation of the NLRP3 inflammasome in microglia: The role of ceramide. J. Neurochem..

[140] Suzuki J., Akahane K., Nakamura J., Naruse K., Kamiya H., Himeno T., Nakamura N., Shibata T., Kondo M., Nagasaki H. (2011). Palmitate induces apoptosis in Schwann cells via both ceramide-dependent and independent pathways. Neuroscience.

[141] Prasad V.V.T.S., Nithipatikom K., Harder D.R. (2008). Ceramide elevates 12-hydroxyeicosatetraenoic acid levels and upregulates 12-lipoxygenase in rat primary hippocampal cell cultures containing predominantly astrocytes. Neurochem. Int..

[142] Darios F., Lambeng N., Troadec J.D., Michel P.P., Ruberg M. (2003). Ceramide increases mitochondrial free calcium levels via caspase 8 and Bid: Role in initiation of cell death. J. Neurochem..

[143] Kleinridders A., Schenten D., Könner A.C., Belgardt B.F., Mauer J., Okamura T., Wunderlich F.T., Medzhitov R., Brüning J.C. (2009). MyD88 signaling in the CNS is required for development of fatty acid-induced leptin resistance and diet-induced obesity. Cell Metab..

[144] Könner A.C., Klöckener T., Brüning J.C. (2009). Control of energy homeostasis by insulin and leptin: Targeting the arcuate nucleus and beyond. Physiol. Behav..

[145] Milanski M., Degasperi G., Coope A., Morari J., Denis R., Cintra D.E., Tsukumo D.M.L., Anhe G., Amaral M.E., Takahashi H.K. (2009). Saturated Fatty Acids Produce an Inflammatory Response Predominantly through the Activation of TLR4 Signaling in Hypothalamus: Implications for the Pathogenesis of Obesity. J. Neurosci..

[146] Tran D.Q., Ramos E.H., Belsham D.D. (2016). Induction of Gnrh mRNA expression by the ω-3 polyunsaturated fatty acid docosahexaenoic acid and the saturated fatty acid palmitate in a GnRH-synthesizing neuronal cell model, mHypoA-GnRH/GFP. Mol. Cell. Endocrinol..

[147] Jang J., Park S., Jin Hur H., Cho H.-J., Hwang I., Pyo Kang Y., Im I., Lee H., Lee E., Yang W. (2016). 25-hydroxycholesterol contributes to cerebral inflammation of X-linked adrenoleukodystrophy through activation of the NLRP3 inflammasome. Nat. Commun..

[148] Le Foll C. (2019). Hypothalamic Fatty Acids and Ketone Bodies Sensing and Role of FAT/CD36 in the Regulation of Food Intake. Front. Physiol..

[149] Tse E.K., Belsham D.D. (2018). Palmitate induces neuroinflammation, ER stress, and Pomc mRNA expression in hypothalamic mHypoA-POMC/GFP neurons through novel mechanisms that are prevented by oleate. Mol. Cell. Endocrinol..

[150] Kwon B., Lee H.-K., Querfurth H.W. (2014). Oleate prevents palmitate-induced mitochondrial dysfunction, insulin resistance and inflammatory signaling in neuronal cells. Biochim. Biophys. Acta-Mol. Cell Res..

[151] McFadden J.W., Aja S., Li Q., Bandaru V.V.R., Kim E.K., Haughey N.J., Kuhajda F.P., Ronnett G.V. (2014). Increasing fatty acid oxidation remodels the hypothalamic neurometabolome to mitigate stress and inflammation. PLoS ONE.

[152] Chen Z., Nie S.-D., Qu M.-L., Zhou D., Wu L.-Y., Shi X.-J., Ma L.-R., Li X., Zhou S.-L., Wang S. (2018). The autophagic degradation of Cav-1 contributes to PA-induced apoptosis and inflammation of astrocytes. Cell Death Dis..

[153] Angelova P.R., Abramov A.Y. (2018). Role of mitochondrial ROS in the brain: From physiology to neurodegeneration. FEBS Lett..

[154] McCann S.K., Roulston C.L. (2013). NADPH Oxidase as a Therapeutic Target for Neuroprotection against Ischaemic Stroke: Future Perspectives. Brain Sci..

[155] Chen H., Kim G.S., Okami N., Narasimhan P., Chan P.H. (2011). NADPH oxidase is involved in post-ischemic brain inflammation. Neurobiol. Dis..

[156] Belarbi K., Cuvelier E., Destée A., Gressier B., Chartier-Harlin M.-C. (2017). NADPH oxidases in Parkinson’s disease: A systematic review. Mol. Neurodegener..

[157] Shimohama S., Tanino H., Kawakami N., Okamura N., Kodama H., Yamaguchi T., Hayakawa T., Nunomura A., Chiba S., Perry G. (2000). Activation of NADPH Oxidase in Alzheimer’s Disease Brains. Biochem. Biophys. Res. Commun..

[158] Maloney E., Sweet I.R., Hockenbery D.M., Pham M., Rizzo N.O., Tateya S., Handa P., Schwartz M.W., Kim F. (2009). Activation of NF-κB by Palmitate in Endothelial Cells. Arterioscler. Thromb. Vasc. Biol..

[159] García-Ruiz I., Solís-Muñoz P., Fernández-Moreira D., Muñoz-Yagüe T., Solís-Herruzo J.A. (2015). In vitro treatment of HepG2 cells with saturated fatty acids reproduces mitochondrial dysfunction found in nonalcoholic steatohepatitis. Dis. Model. Mech..

[160] Farmer B.C., Walsh A.E., Kluemper J.C., Johnson L.A. (2020). Lipid Droplets in Neurodegenerative Disorders. Front. Neurosci..

[161] Le Foll C., Levin B.E. (2016). Fatty acid-induced astrocyte ketone production and the control of food intake. Am. J. Physiol.-Regul. Integr. Comp. Physiol..

[162] Couvineau A., Voisin T., Nicole P., Gratio V., Abad C., Tan Y.V. (2019). Orexins as Novel Therapeutic Targets in Inflammatory and Neurodegenerative Diseases. Front. Endocrinol..

[163] Kumar P., Kumar A. (2008). Prolonged pretreatment with carvedilol prevents alterations and oxidative stress in rats. Pharmacol. Rep..

[164] Maciejczyk M., Żebrowska E., Zalewska A., Chabowski A. (2018). Redox Balance, Antioxidant Defense, and Oxidative Damage in the Hypothalamus and Cerebral Cortex of Rats with High Fat Diet-Induced Insulin Resistance. Oxid. Med. Cell. Longev..

[165] Hetz C., Glimcher L.H. (2009). Fine-tuning of the unfolded protein response: Assembling the IRE1alpha interactome. Mol. Cell.

[166] Hetz C., Mollereau B. (2014). Disturbance of endoplasmic reticulum proteostasis in neurodegenerative diseases. Nat. Rev. Neurosci..

[167] Hotamisligil G.S. (2010). Endoplasmic reticulum stress and the inflammatory basis of metabolic disease. Cell.

[168] Santos L.E., Ferreira S.T. (2018). Crosstalk between endoplasmic reticulum stress and brain inflammation in Alzheimer’s disease. Neuropharmacology.

[169] Chiti F., Dobson C.M. (2017). Protein Misfolding, Amyloid Formation, and Human Disease: A Summary of Progress over the Last Decade. Annu. Rev. Biochem..

[170] Lindholm D., Wootz H., Korhonen L. (2006). ER stress and neurodegenerative diseases. Cell Death Differ..

[171] Kim J., Park Y.-J., Jang Y., Kwon Y.H. (2011). AMPK activation inhibits apoptosis and tau hyperphosphorylation mediated by palmitate in SH-SY5Y cells. Brain Res..

[172] Yamaguchi Y., Miura M. (2015). Programmed Cell Death in Neurodevelopment. Dev. Cell.

[173] Ulloth J.E., Casiano C.A., De Leon M. (2003). Palmitic and stearic fatty acids induce caspase-dependent and -independent cell death in nerve growth factor differentiated PC12 cells. J. Neurochem..

[174] Yuan Q., Zhao S., Wang F., Zhang H., Chen Z.-J., Wang J., Wang Z., Du Z., Ling E.-A., Liu Q. (2013). Palmitic acid increases apoptosis of neural stem cells via activating c-Jun N-terminal kinase. Stem Cell Res..

[175] Nikoletopoulou V., Papandreou M.-E., Tavernarakis N. (2014). Autophagy in the physiology and pathology of the central nervous system. Cell Death Differ..

[176] Montero M.L., Liu J.W., Orozco J., Casiano C.A., De Leon M. (2020). Docosahexaenoic acid protection against palmitic acid-induced lipotoxicity in NGF-differentiated PC12 cells involves enhancement of autophagy and inhibition of apoptosis and necroptosis. J. Neurochem..

[177] Bylicky M.A., Mueller G.P., Day R.M. (2018). Mechanisms of endogenous neuroprotective effects of astrocytes in brain injury. Oxid. Med. Cell. Longev..

[178] Ortiz-Rodriguez A., Arevalo M.A. (2020). The contribution of astrocyte autophagy to systemic metabolism. Int. J. Mol. Sci..

[179] Fatima S., Hu X., Gong R.-H., Huang C., Chen M., Wong H.L.X., Bian Z., Kwan H.Y. (2019). Palmitic acid is an intracellular signaling molecule involved in disease development. Cell. Mol. Life Sci..

[180] Shah A., Han P., Wong M.-Y., Chang C.R., Legido-Quigley C. (2019). Palmitate and Stearate are Increased in the Plasma in a 6-OHDA Model of Parkinson’s Disease. Metabolites.

[181] Valdearcos M., Robblee M.M., Benjamin D.I., Nomura D.K., Xu A.W., Koliwad S.K. (2017). Microglia dictate the impact of saturated fat consumption on hypothalamic inflammation and neuronal function. Cell Rep..

[182] Cakir I., Nillni E.A. (2019). Endoplasmic Reticulum Stress, the Hypothalamus, and Energy Balance. Trends Endocrinol. Metab..

[183] Shamim A., Mahmood T., Ahsan F., Kumar A., Bagga P. (2018). Lipids: An insight into the neurodegenerative disorders. Clin. Nutr. Exp..

[184] Reitz C., Mayeux R. (2014). Alzheimer disease: Epidemiology, diagnostic criteria, risk factors and biomarkers. Biochem. Pharmacol..

[185] Fargo K.N., Aisen P., Albert M., Au R., Corrada M.M., DeKosky S., Drachman D., Fillit H., Gitlin L., Haas M. (2014). 2014 Report on the Milestones for the US National Plan to Address Alzheimer’s Disease. Alzheimers Dement..

[186] Desale S.E., Chinnathambi S. (2020). Role of dietary fatty acids in microglial polarization in Alzheimer’s disease. J. Neuroinflammation.

[187] Alonso A.D., Cohen L.S., Corbo C., Morozova V., ElIdrissi A., Phillips G., Kleiman F.E. (2018). Hyperphosphorylation of Tau Associates With Changes in Its Function Beyond Microtubule Stability. Front. Cell. Neurosci..

[188] Brunello C.A., Merezhko M., Uronen R.-L., Huttunen H.J. (2020). Mechanisms of secretion and spreading of pathological tau protein. Cell. Mol. Life Sci..

[189] Marwarha G., Claycombe-Larson K., Lund J., Schommer J., Ghribi O. (2019). A Diet Enriched in Palmitate and Deficient in Linoleate Exacerbates Oxidative Stress and Amyloid-β Burden in the Hippocampus of 3xTg-AD Mouse Model of Alzheimer’s Disease. J. Alzheimers Dis..

[190] Edwards Iii G.A., Gamez N., Escobedo G., Calderon O., Moreno-Gonzalez I. (2019). Modifiable Risk Factors for Alzheimer’s Disease. Front. Aging Neurosci..

[191] Hashimoto S., Saido T.C. (2018). Critical review: Involvement of endoplasmic reticulum stress in the aetiology of Alzheimer’s disease. Open Biol..

[192] Nasaruddin M.L., Hölscher C., Kehoe P., Graham S.F., Green B.D. (2016). Wide-ranging alterations in the brain fatty acid complement of subjects with late Alzheimer’s disease as detected by GC-MS. Am. J. Transl. Res..

[193] Kim J.Y., Lee H.J., Lee S.-J., Jung Y.H., Yoo D.Y., Hwang I.K., Seong J.K., Ryu J.M., Han H.J. (2017). Palmitic Acid-BSA enhances Amyloid-β production through GPR40-mediated dual pathways in neuronal cells: Involvement of the Akt/mTOR/HIF-1α and Akt/NF-κB pathways. Sci. Rep..

[194] Bhattacharyya R., Barren C., Kovacs D.M. (2013). Palmitoylation of amyloid precursor protein regulates amyloidogenic processing in lipid rafts. J. Neurosci..

[195] Liu L., Martin R., Kohler G., Chan C. (2013). Palmitate induces transcriptional regulation of BACE1 and presenilin by STAT3 in neurons mediated by astrocytes. Exp. Neurol..

[196] Amtul Z., Keet M., Wang L., Merrifield P., Westaway D., Rozmahel R.F. (2011). DHA Supplemented in Peptamen Diet Offers No Advantage in Pathways to Amyloidosis: Is It Time to Evaluate Composite Lipid Diet?. PLoS ONE.

[197] Connolly B.S., Lang A.E. (2014). Pharmacological Treatment of Parkinson Disease: A Review. JAMA.

[198] Armstrong M.J., Okun M.S. (2020). Diagnosis and Treatment of Parkinson Disease: A Review. JAMA.

[199] Alecu I., Bennett S.A.L. (2019). Dysregulated lipid metabolism and its role in α-synucleinopathy in Parkinson’s disease. Front. Neurosci..

[200] Xicoy H., Wieringa B., Martens G.J.M. (2019). The Role of Lipids in Parkinson’s Disease. Cells.

[201] Fields C.R., Bengoa-Vergniory N., Wade-Martins R. (2019). Targeting Alpha-Synuclein as a Therapy for Parkinson’s Disease. Front. Mol. Neurosci..

[202] Gonzalez-Riano C., Saiz J., Barbas C., Bergareche A., Huerta J.M., Ardanaz E., Konjevod M., Mondragon E., Erro M.E., Chirlaque M.D. (2021). Prognostic biomarkers of Parkinson’s disease in the Spanish EPIC cohort: A multiplatform metabolomics approach. NPJ Park. Dis..

[203] Castagnet P.I., Golovko M.Y., Barceló-Coblijn G.C., Nussbaum R.L., Murphy E.J. (2005). Fatty acid incorporation is decreased in astrocytes cultured from α-synuclein gene-ablated mice. J. Neurochem..

[204] Su X., Chu Y., Kordower J.H., Li B., Cao H., Huang L., Nishida M., Song L., Wang D., Federoff H.J. (2015). PGC-1α promoter methylation in Parkinson’s disease. PLoS ONE.

[205] Angelova P.R., Esteras N., Abramov A.Y. (2020). Mitochondria and lipid peroxidation in the mechanism of neurodegeneration: Finding ways for prevention. Med. Res. Rev..

[206] Hayes C.E., Ntambi J.M. (2020). Multiple Sclerosis: Lipids, Lymphocytes, and Vitamin D. Immunometabolism.

[207] Armon-Omer A., Waldman C., Simaan N., Neuman H., Tamir S., Shahien R. (2019). New Insights on the Nutrition Status and Antioxidant Capacity in Multiple Sclerosis Patients. Nutrients.

[208] Nogueras L., Gonzalo H., Jové M., Sol J., Gil-Sanchez A., Hervás J.V., Valcheva P., Gonzalez-Mingot C., Solana M.J., Peralta S. (2019). Lipid profile of cerebrospinal fluid in multiple sclerosis patients: A potential tool for diagnosis. Sci. Rep..

[209] Sturrock A., Leavitt B.R. (2010). The clinical and genetic features of Huntington disease. J. Geriatr. Psychiatry Neurol..

[210] Butland S.L., Sanders S.S., Schmidt M.E., Riechers S.-P.P., Lin D.T.S.S., Martin D.D.O.O., Vaid K., Graham R.K., Singaraja R.R., Wanker E.E. (2014). The palmitoyl acyltransferase HIP14 shares a high proportion of interactors with huntingtin: Implications for a role in the pathogenesis of Huntington’s disease. Hum. Mol. Genet..

[211] Baldwin A.C., Green C.D., Olson L.K., Moxley M.A., Corbett J.A. (2012). A role for aberrant protein palmitoylation in FFA-induced ER stress and β-cell death. Am. J. Physiol. Endocrinol. Metab..

[212] Gunaratnam K., Vidal C., Gimble J.M., Duque G. (2014). Mechanisms of palmitate-induced lipotoxicity in human osteoblasts. Endocrinology.

[213] Kang R., Wang L., Sanders S.S., Zuo K., Hayden M.R., Raymond L.A. (2019). Altered Regulation of Striatal Neuronal N-Methyl-D-Aspartate Receptor Trafficking by Palmitoylation in Huntington Disease Mouse Model. Front. Synaptic Neurosci..

[214] Tang K.S. (2014). Protective effect of arachidonic acid and linoleic acid on 1-methyl-4-phenylpyridinium-induced toxicity in PC12 cells. Lipids Health Dis..

[215] Marcheselli V.L., Hong S., Lukiw W.J., Tian X.H., Gronert K., Musto A., Hardy M., Gimenez J.M., Chiang N., Serhan C.N. (2003). Novel Docosanoids Inhibit Brain Ischemia-Reperfusion-mediated Leukocyte Infiltration and Pro-inflammatory Gene Expression. J. Biol. Chem..

[216] Meng Q., Luchtman D.W., El Bahh B., Zidichouski J.A., Yang J., Song C. (2010). Ethyl-eicosapentaenoate modulates changes in neurochemistry and brain lipids induced by parkinsonian neurotoxin 1-methyl-4-phenylpyridinium in mouse brain slices. Eur. J. Pharmacol..

[217] Luchtman D.W., Meng Q., Song C. (2012). Ethyl-eicosapentaenoate (E-EPA) attenuates motor impairments and inflammation in the MPTP-probenecid mouse model of Parkinson’s disease. Behav. Brain Res..

[218] Luchtman D.W., Meng Q., Wang X., Shao D., Song C. (2013). Omega-3 fatty acid eicospentaenoic acid attenuates MPP+-induced neurodegeneration in fully differentiated human SH-SY5Y and primary mesencephalic cells. J. Neurochem..

[219] Moazedi A.A., Hossienzadeh Z., Chinpardaz R. (2007). The effects of coadministration palmitic acid and oleic acid (omega 9) on spatial learning and motor activity in adult male rat. Pakistan J. Biol. Sci..

[220] Rozpędek-Kamińska W., Siwecka N., Wawrzynkiewicz A., Wojtczak R., Pytel D., Diehl J.A., Majsterek I. (2020). The PERK-dependent molecular mechanisms as a novel therapeutic target for neurodegenerative diseases. Int. J. Mol. Sci..

[221] Raefsky S.M., Furman R., Milne G., Pollock E., Axelsen P., Mattson M.P., Shchepinov M.S. (2018). Deuterated polyunsaturated fatty acids reduce brain lipid peroxidation and hippocampal amyloid β-peptide levels, without discernable behavioral effects in an APP/PS1 mutant transgenic mouse model of Alzheimer’s disease. Neurobiol. Aging.

[222] Elharram A., Czegledy N.M., Golod M., Milne G.L., Pollock E., Bennett B.M., Shchepinov M.S. (2017). Deuterium-reinforced polyunsaturated fatty acids improve cognition in a mouse model of sporadic Alzheimer’s disease. FEBS J..

[223] Ma E., Ingram K.H., Milne G.L., Garvey W.T. (2017). F2-Isoprostanes Reflect Oxidative Stress Correlated with Lean Mass and Bone Density but Not Insulin Resistance. J. Endocr. Soc..

[224] Famitafreshi H., Karimian M. (2020). Prostaglandins as the Agents That Modulate the Course of Brain Disorders. Degener. Neurol. Neuromuscul. Dis..

[225] Joffre C., Dinel A.L., Chataigner M., Pallet V., Layé S. (2020). N-3 polyunsaturated fatty acids and their derivates reduce neuroinflammation during aging. Nutrients.

[226] Lafourcade M., Larrieu T., Mato S., Duffaud A., Sepers M., Matias I., De Smedt-Peyrusse V., Labrousse V.F., Bretillon L., Matute C. (2011). Nutritional omega-3 deficiency abolishes endocannabinoid-mediated neuronal functions. Nat. Neurosci..

[227] Larrieu T., Madore C., Joffre C., Layé S. (2012). Nutritional n-3 polyunsaturated fatty acids deficiency alters cannabinoid receptor signaling pathway in the brain and associated anxiety-like behavior in mice. J. Physiol. Biochem..

[228] Wu A., Ying Z., Gomez-Pinilla F. (2004). Dietary Omega-3 Fatty Acids Normalize BDNF Levels, Reduce Oxidative Damage, and Counteract Learning Disability after Traumatic Brain Injury in Rats. J. Neurotrauma.

[229] Rao J.S., Ertley R.N., Lee H.-J., DeMar Jr J.C., Arnold J.T., Rapoport S.I., Bazinet R.P. (2007). n-3 polyunsaturated fatty acid deprivation in rats decreases frontal cortex BDNF via a p38 MAPK-dependent mechanism. Mol. Psychiatry.

[230] Gómez-Soler M., Cordobilla B., Morató X., Fernández-Dueñas V., Domingo J.C., Ciruela F. (2018). Triglyceride Form of Docosahexaenoic Acid Mediates Neuroprotection in Experimental Parkinsonism. Front. Neurosci..

[231] Kramer M., Mironova E., Vanilovich I., Platt R., Matush L., Igumnov S., Fombonne E., Bogdanovich N., Collet J.-P., Chalmers B. (2008). Breastfeeding and child cognitive development. Child Care Health Dev..

[232] Cooper R.E., Tye C., Kuntsi J., Vassos E., Asherson P. (2015). Omega-3 polyunsaturated fatty acid supplementation and cognition: A systematic review and meta-analysis. J. Psychopharmacol..

[233] Witte A.V., Kerti L., Hermannstädter H.M., Fiebach J.B., Schreiber S.J., Schuchardt J.P., Hahn A., Flöel A. (2014). Long-Chain Omega-3 Fatty Acids Improve Brain Function and Structure in Older Adults. Cereb. Cortex.

[234] Fitzgerald K.C., O’Reilly É.J., Falcone G.J., McCullough M.L., Park Y., Kolonel L.N., Ascherio A. (2014). Dietary ω-3 polyunsaturated fatty acid intake and risk for amyotrophic lateral sclerosis. JAMA Neurol..

[235] Ali W., Ikram M., Park H.Y., Jo M.G., Ullah R., Ahmad S., Abid N.B., Kim M.O. (2020). Oral Administration of Alpha Linoleic Acid Rescues Aβ-Induced Glia-Mediated Neuroinflammation and Cognitive Dysfunction in C57BL/6N Mice. Cells.

[236] Piomelli D. (2013). A fatty gut feeling. Trends Endocrinol. Metab..

[237] Thevenet J., De Marchi U., Domingo J.S., Christinat N., Bultot L., Lefebvre G., Sakamoto K., Descombes P., Masoodi M., Wiederkehr A. (2016). Medium-chain fatty acids inhibit mitochondrial metabolism in astrocytes promoting astrocyte–neuron lactate and ketone body shuttle systems. FASEB J..

[238] Lalwani A.M., Yilmaz A., Bisgin H., Ugur Z., Akyol S., Graham S.F. (2020). The biochemical profile of post-mortem brain from people who suffered from epilepsy reveals novel insights into the etiopathogenesis of the disease. Metabolites.

[239] Kao Y.C., Ho P.C., Tu Y.K., Jou I.M., Tsai K.J. (2020). Lipids and Alzheimer’s disease. Int. J. Mol. Sci..

[240] Page K.A., Williamson A., Yu N., McNay E.C., Dzuira J., McCrimmon R.J., Sherwin R.S. (2009). Medium-chain fatty acids improve cognitive function in intensively treated type 1 diabetic patients and support in vitro synaptic transmission during acute hypoglycemia. Diabetes.

[241] Zhao W., Varghese M., Vempati P., Dzhun A., Cheng A., Wang J., Lange D., Bilski A., Faravelli I., Pasinetti G.M. (2012). Caprylic Triglyceride as a Novel Therapeutic Approach to Effectively Improve the Performance and Attenuate the Symptoms Due to the Motor Neuron Loss in ALS Disease. PLoS ONE.

[242] Wein S., Wolffram S., Schrezenmeir J., Gašperiková D., Klimeš I., Šeböková E. (2009). Medium-chain fatty acids ameliorate insulin resistance caused by high-fat diets in rats. Diabetes Metab. Res. Rev..

[243] Haghikia A., Jörg S., Duscha A., Berg J., Manzel A., Waschbisch A., Hammer A., Lee D.H., May C., Wilck N. (2015). Dietary Fatty Acids Directly Impact Central Nervous System Autoimmunity via the Small Intestine. Immunity.

